# Supplementary material for: Differentiation-state plasticity is a targetable resistance mechanism in basal-like breast cancer
Source: Nat Commun. 2018 Sep 19;9:3815. doi: 10.1038/s41467-018-05729-w (PMC6145927; doi:10.1038/s41467-018-05729-w)
Supplement: Supplementary file 1 — Supplementary Information [file 41467_2018_5729_MOESM1_ESM.pdf]

Differentiation-state plasticity is a targetable resistance mechanism in basal-like breast cancer

Risom et al.

Supplementary Information

Pg. 2-25 Supplementary Figures 1-15
Pg. 26-28 Supplementary Tables 1 and 2
Pg. 29-33 Supplementary Methods

Supplementary Figure 1 | Measuring differentiation-state heterogeneity in primary breast tumors and patient-derived xenograft tumors of different histological subtype.

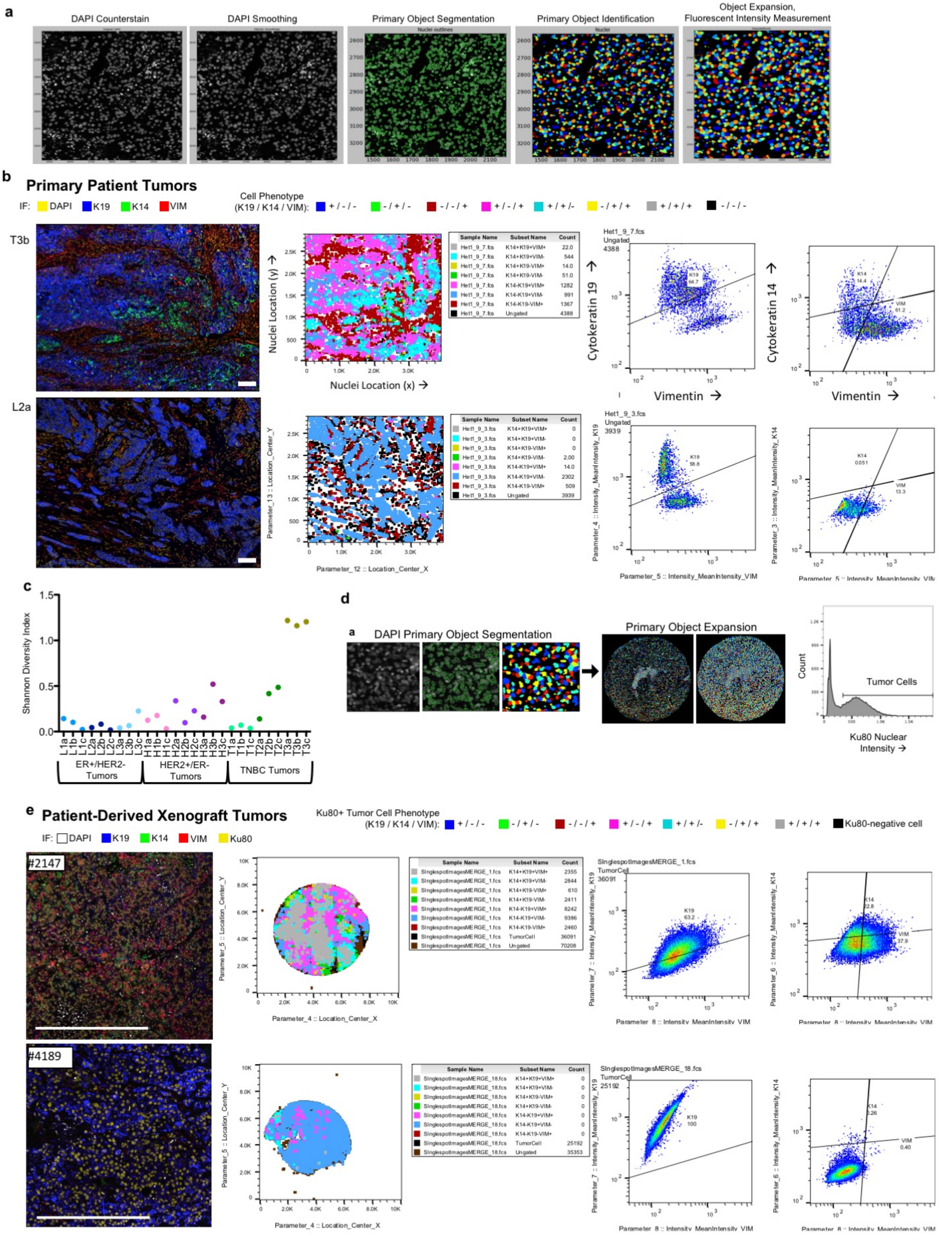

Supplementary Figure 1. Measuring differentiation-state heterogeneity in primary breast tumors and patient-derived xenograft tumors of different histological subtype.

(**a**) Schematic showing the Cell Profiler workflow to measure single cell intensity of cell-state marker expression in 4-color-IF images of FFPE primary tumor samples. DAPI signal is smoothed and single nuclei are segmented, and an expanded annulus from each nucleus is used to measure signal intensity from the 488, 568, and 647 channels. (**b**) Two examples of primary patient tumors and the resulting analyses to determine single cell phenotype. Left panel: 4-color IF images of treatment-naïve breast tumors showing DAPI (yellow), K19 (blue), K14 (green) and VIM (red). Middle panel: An x/y dotplot showing single cell location and cell phenotype by color, with an accompanying legend with phenotype counts. Right Panel: dot plots comparing single cell intensity of K19 vs. VIM, and K14 vs. VIM with positivity gates shown. Tumor name is given to the left of the IF image, different regions of the same tumor are denoted by letter e.g. T3a, T3b, T3c. See Supplementary Dataset 1 for all additional tumors; T = TNBC, L = Luminal (ER+/PR+). Scale bars = 100μm. (**c**) The Shannon diversity index was calculated using K19/K14/VIM differentiation-state frequencies in each tumor and displayed, with tumors grouped by histological subtype. Color denotes individual tumors, with three regions of each tumor analyzed. (**d**) Schematic showing the Cell Profiler workflow to measure single cell intensity of cell-state marker expression in 4-color-IF images of patient-derived xenograft tissue microarray spots, Ku80 gating is used to identify human tumor cells. (**e**) Two examples of patient-derived xenograft tumors and the resulting analyses to determine single cell phenotype. Left panel: 4-color IF images of patient-derived xenograft tumors showing DAPI (yellow), K19 (blue), K14 (green) and VIM (red), scale bars = 100μm. Middle panel: An x/y dotplot showing single cell location and cell phenotype by color in each tissue-microarray spot analyzed, with an accompanying legend with phenotype counts. Only human Ku80+ tumor cells were analyzed for K19/K14/VIM positivity, non-tumor cells are shown in black. Right Panel: dot plots comparing single tumor cell intensity of K19 vs. VIM, and K14 vs. VIM, with positivity gates shown.

Supplementary Figure 2 | Interrogating differentiation state heterogeneity in breast cancer cell lines.

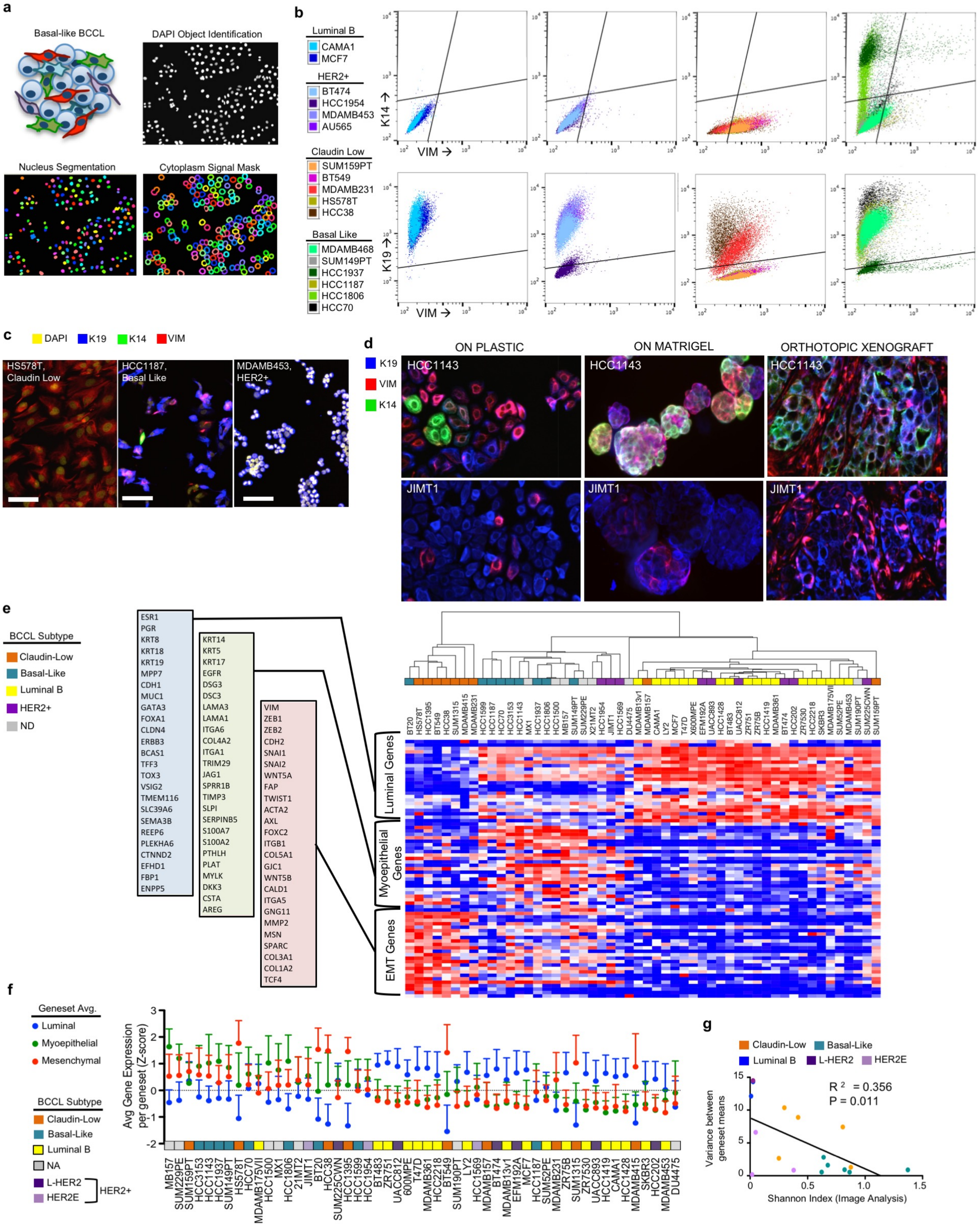

Supplementary Figure 2. Interrogating differentiation state heterogeneity in breast cancer cell lines. (**a**) Schematic showing the Cell Profiler workflow to measure single cell intensity of cell-state marker expression in 4-color-IF images of breast cancer cell lines. DAPI signal is used to segment single nuclei, and an expanded annulus from each nucleus (not including nuclear signal) is used to measure signal intensity from the 488, 568, and 647 channels in the cell cytoplasm. (**b**) Image cytometry plots showing K14 vs VIM expression, and K19 vs VIM expression, for multiple luminal B, claudin-low, HER2E, and basal-like cell lines. K19+, K14+ and VIM+ gates displayed. (**c**) Images of three cell lines (in addition to Fig. 1h) of different molecular subtypes showing DAPI (yellow), K14 (green) and VIM (red). Scale bars = 100µm. (**d**) Images of HCC1143 (basal-like) and JIMT1 (HER2E) cells grown in 2D on plastic, grown on top of matrigel, and grown as orthotopic xenografts, showing K19 (blue), K14 (green) and VIM (red). (**e**) Heatmap of gene expression showing 45 breast cancer cell lines and their expression of 25 luminal, 25 myoepithelial, and 25 mesenchymal-transition correlated genes as shown in Figure 2a with the addition of cell line and gene names, cell lines arranged by unsupervised Pearson-coefficient clustering. Cell line molecular subtype is denoted by color: claudin-low (orange), basal-like (cyan), luminal B (yellow), HER2E (purple), not determined (ND, white). (**f**) Graph of the mean normalized gene expression value (Z-score) of the luminal (L, blue), basal (B, green), and mesenchymal (M, red) genesets are displayed in each cell line with standard deviation of expression. Cell lines are arranged in descending order according to the mean Z-score of all genesets. Cell line subtype is denoted by color as in ***e***. (**g**) Scatterplot comparing the geneset variance metric from transcriptional data (Y axis) to the Shannon diversity indices from imaging data (X axis) with accompanying linear regression analysis. Regression fit (R^2) and significance of non-zero slope (P-value) are displayed.

Supplementary Figure 3 | Targeted therapy drug screen in SUM149PT

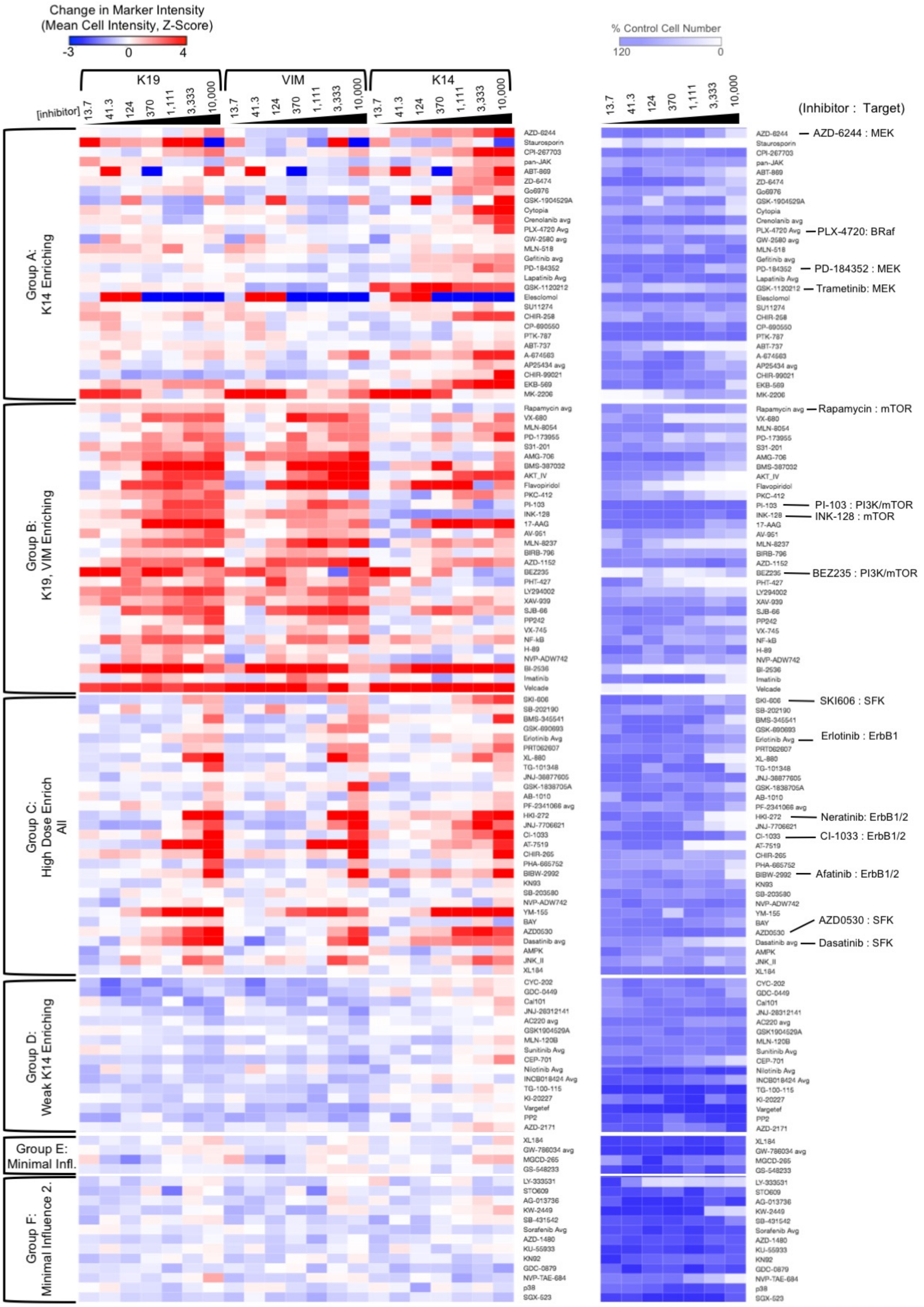

Supplementary Figure 3. Targeted therapy drug screen in SUM149PT.
Heatmap showing the results of a 72hr drug screen with 7-doses of 119 targeted therapeutics in SUM149PT cells, examining % control viability (right) and the change in K19, K14 and VIM expression compared to DMSO control wells (Z-score). Drugs are arranged and grouped by K=6 K-means clustering with all drugs labeled. Select drugs that are highlighted in ***Figure 2a*** have enlarged bold labels.

Supplementary Figure 4 | MEK and PI3K/mTOR inhibitors have an opposing influence on the frequency of basal-differentiated cells in drug-persisting populations.

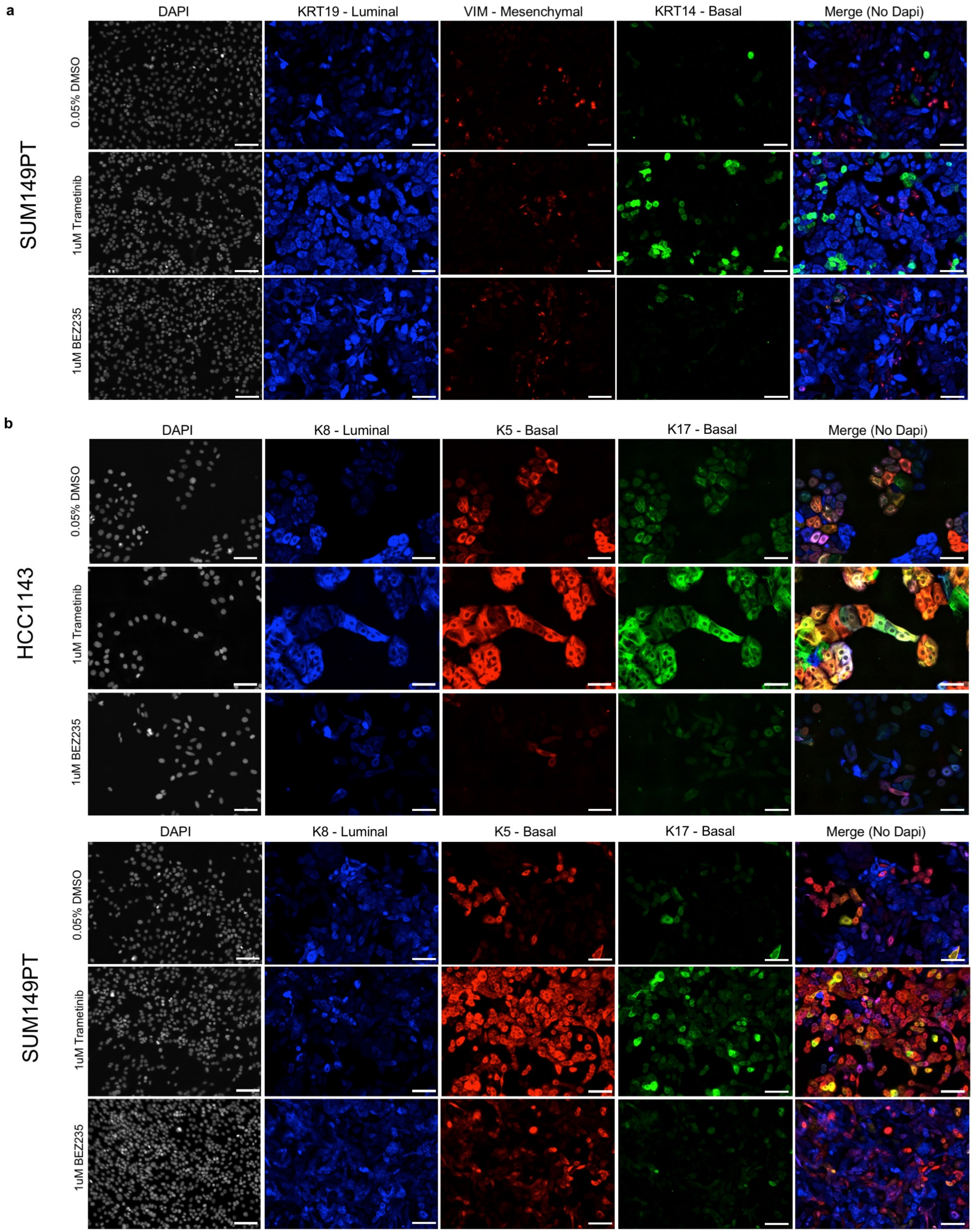

Supplementary Figure 4. MEK and PI3K/mTOR inhibitors have an opposing influence on the frequency of basal-differentiated cells in drug-persisting populations.
(**a**) Images of SUM149PT under DMSO control conditions or following 72hr of 1μM Trametinib or 1μM BEZ235, showing DAPI, K19, VIM, K14 and a 4-color merged image, scale bars = 100μm (**b**) Images of HCC1143 and SUM149PT under DMSO control conditions or following 72hr of 1μM Trametinib of 1μM BEZ235, showing DAPI, K8, K5, K17 and a 4-color merged image, scale bars = 100μm.

Supplementary Figure 5 | MEK and PI3K/mTOR enrich distinct DTP differentiation-states in basal-like cell lines.

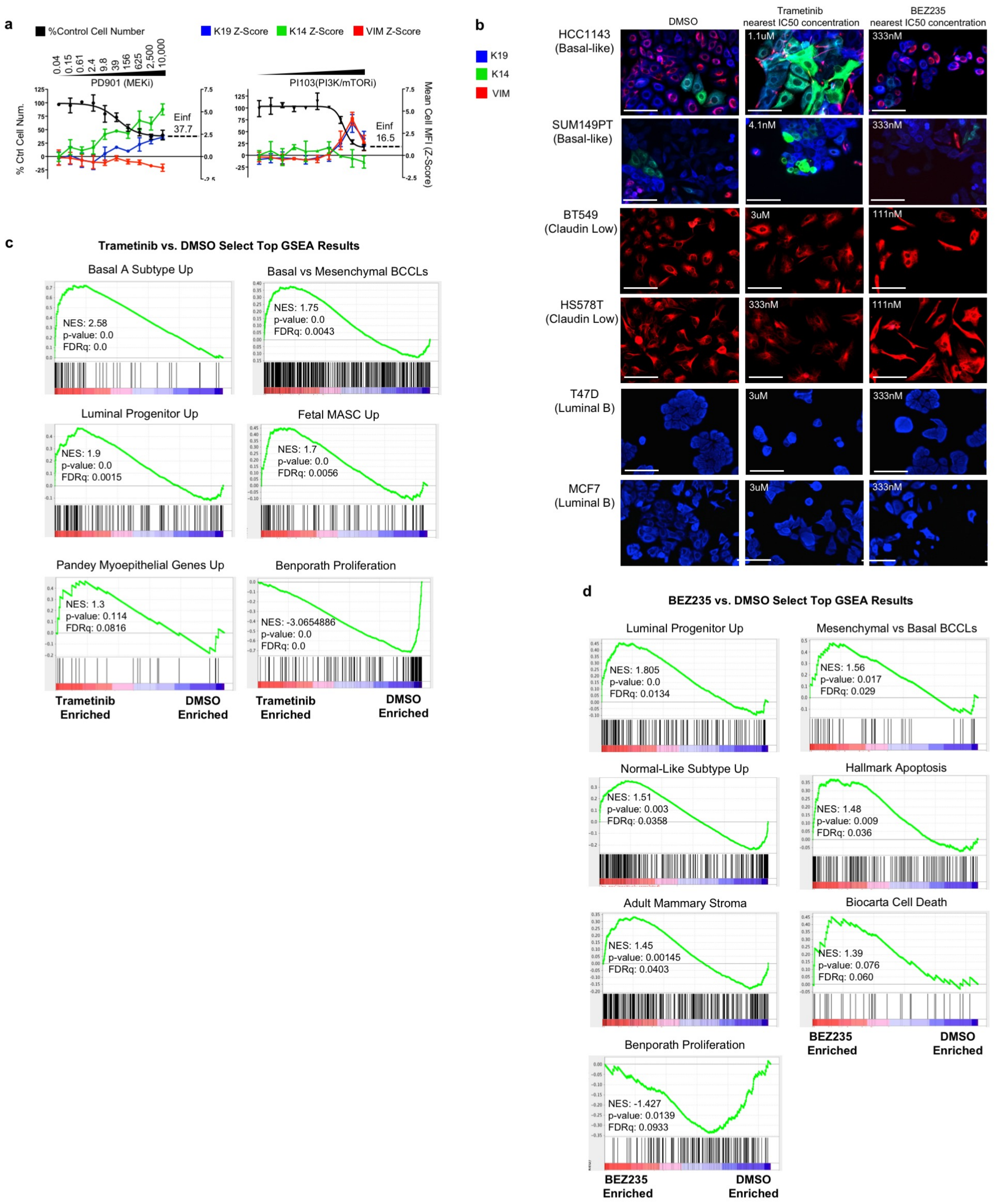

Supplementary Figure 5. MEK and PI3K/mTOR inhibitors enrich distinct DTP differentiation-states in basal-like cell lines.

(**a**) Graphs of therapy-induced changes in cell number (left axis) and mean-cell MFI (right axis, as Z-score) of K19 (blue), VIM (red), and K14 (green), in HCC1143 cells following 72hr incubation with increasing doses of the MEK inhibitor PD901, or PI3K/mTOR inhibitor PI103. The projected maximum level of inhibition, or Einf, is shown for each drug. (**b**) Images of two basal-like cell lines (HCC1143 and SUM149PT), two Claudin-low lines (BT549 and HS578T), or two Luminal B cell lines (T47D and MCF7) following 72h treatment with the nearest-to-IC50 dose of Trametinib, BEZ235, or DMSO, dose displayed on image. K19 (blue), VIM (red), and K14 (green) shown, scale bars = 100μm. (**c**) Select top-enriched genesets are shown for 6day Trametinib treated HCC1143 cells vs. DMSO with Normalized Enrichment Score (NES), Nominal P-value (p-value), and FDR q-value (FDRq) shown. (**d**) Select top-enriched genesets are shown for 6day BEZ235 treated HCC1143 cells vs. DMSO.

Supplementary Figure 6 | Further characterization of Trametinib and BEZ235 DTP phenotypes.

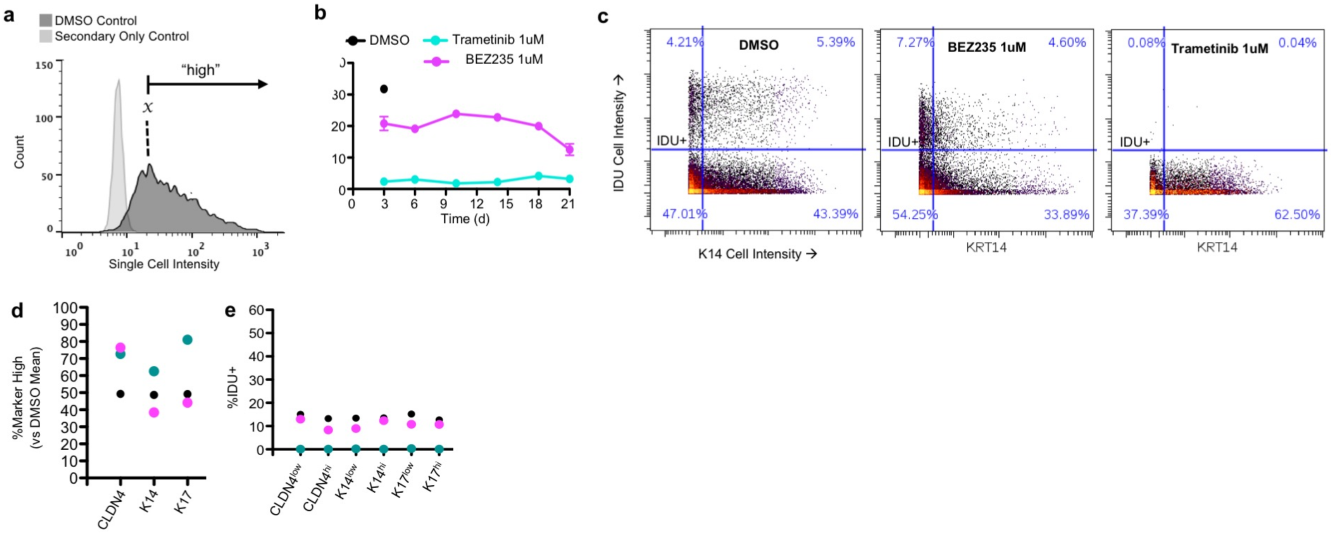

Supplementary Figure 6. Further characterization of Trametinib and BEZ235 DTP phenotypes. (**a**) Schematic showing how high expression of a phenotype marker is determined from DMSO population values. (**b**) Graph showing EdU incorporation rates in HCC1143 cells every 3-4 days while being maintained on 1μM Trametinib or 1μM BEZ235 for 21 days, with the incorporation rate of DMSO shown for the first 3 days of growth. (**c**) Dot plots showing mass cytometry results comparing single cell IdU and K14 levels following 72hr of 1μM Trametinib, 1μM BEZ235, or a DMSO control. (**d**) Graph showing the percent of cells expressing high levels of the luminal marker CLDN4, basal markers K14 and K17, or mesenchymal marker VIM following 72hr of 1μM Trametinib or BEZ235, measured by mass cytometry. (**e**) Graph showing the frequency of IdU+ cells in cells also expressing high and low values of CLDN4, K14, K17, and VIM from ***d.***

Supplementary Figure 7 | Assessing genotypic selection following Trametinib and BEZ235.

a

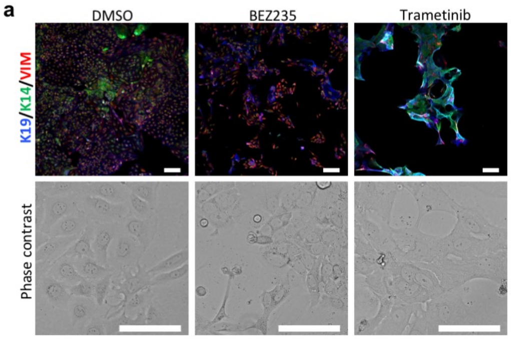

b

Replicate Library	Treatment	Mean Coverage	% On-Target	% Duplication
dmso_rep1.pe.sort.bam	DMSO	113	0.56	0.11
bez235_rep1.pe.sort.bam	BEZ235	115	0.59	0.12
trametinib_rep1.pe.sort.bam	Trametinib	100	0.57	0.11
dmso_rep2.pe.sort.bam	DMSO	131	0.53	0.12
bez235_rep2.pe.sort.bam	BEZ235	96	0.58	0.10
trametinib_rep2.pe.sort.bam	Trametinib	103	0.58	0.11
dmso_rep3.pe.sort.bam	DMSO	112	0.61	0.11
bez235_rep3.pe.sort.bam	BEZ235	93	0.52	0.08
trametinib_rep3.pe.sort.bam	Trametinib	99	0.56	0.10

c

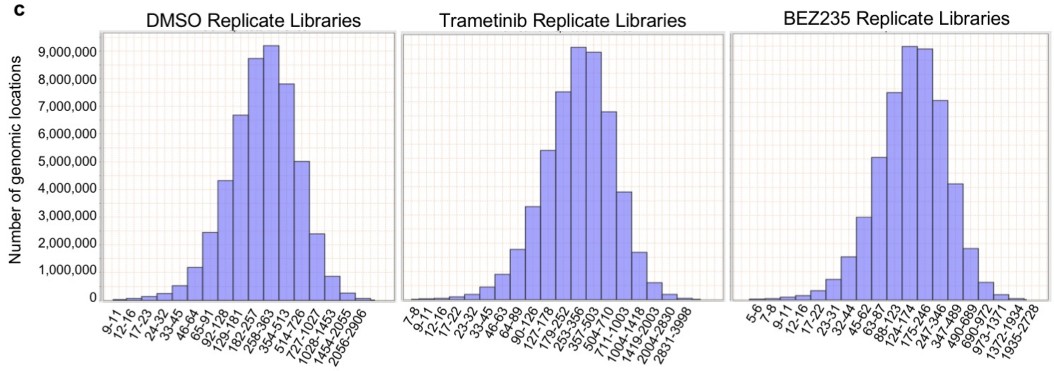

d

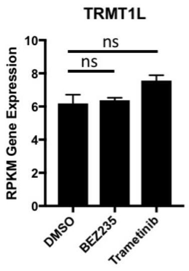

e

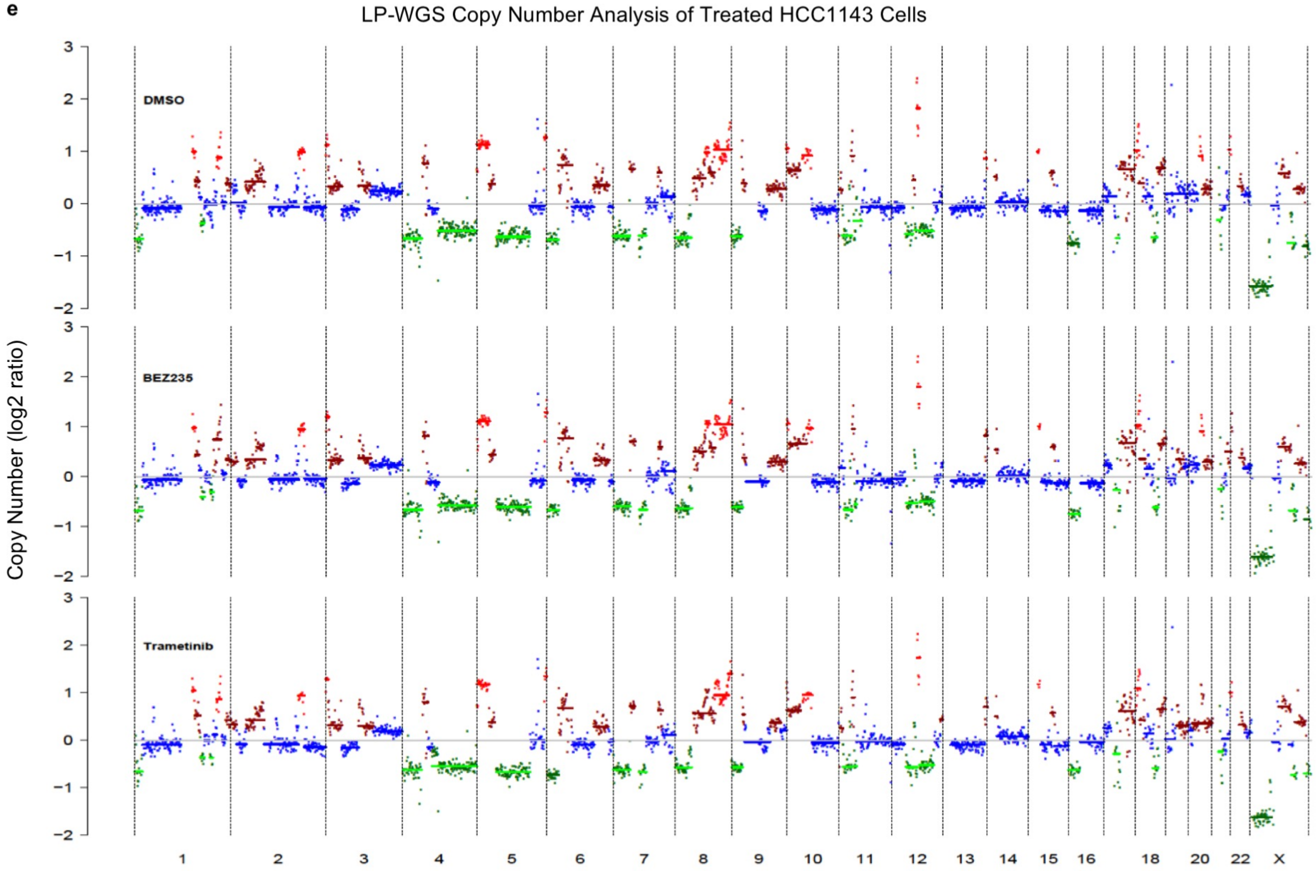

Supplementary Figure 7. Assessing genomic selection following Trametinib or BEZ235.
(**a**) The phenotypic change in HCC1143 cells that were harvested for whole exome sequencing and whole genome sequencing analysis is shown by three color IF with K19 (blue), K14 (green) and VIM (red) as well as with phase contrast, scale bars = 100μm. (**b**) A table showing mean sequencing statistics for each experimental replicate from WES analysis. (**c**) Histograms showing the distribution of sequencing coverage by genomic location for the different experimental conditions (pooled replicates). (**d**) Gene expression of TRMT1L is compared between treatment groups. (**e**) Copy number alteration plots of HCC1143 cells under the various treatment conditions, where blue represents copy neutral, green represents deletion, maroon represents gain, and red represents amplification.

Supplementary Figure 8 | Computational modeling of the BEZ235 response and drug washout analysis.

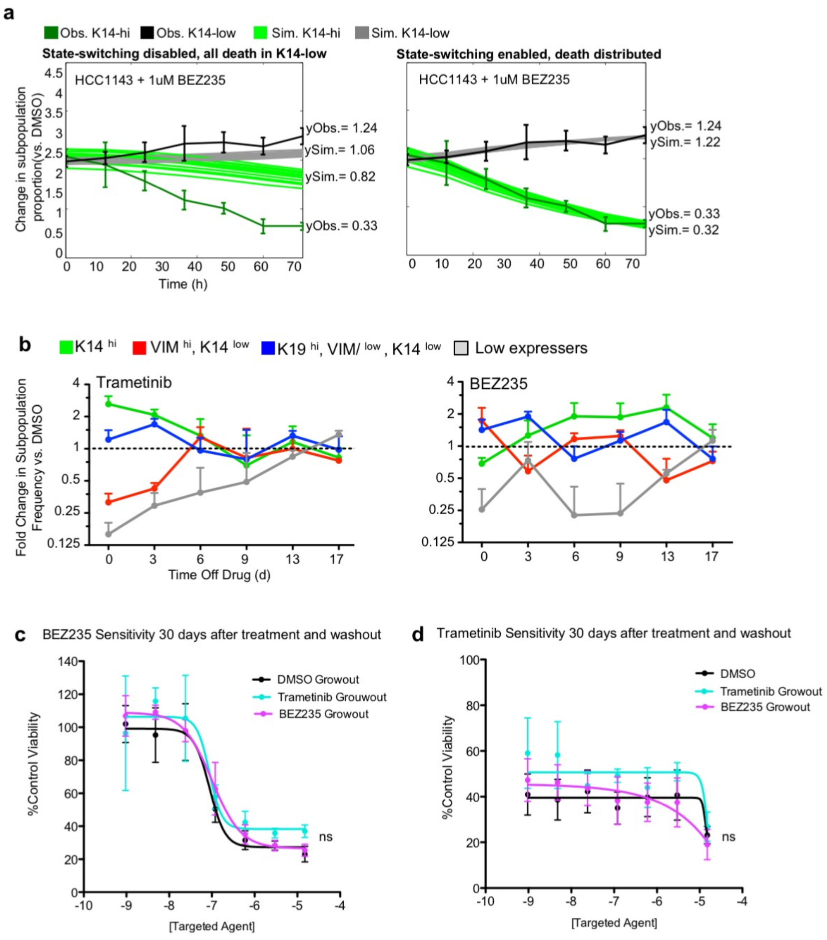

Supplementary Figure 8. Computational modeling of the BEZ235 response and drug washout analysis. (**a**) Simulated fold change of 1µM BEZ235-treated HCC1143 cells vs. DMSO using the model presented in Fig. 3g with two separate hypotheses: *K14^low^ Darwinian selection* (left) where all cell death occurs in K14^{hi} cells and cells cannot transition between states, *cell-state transition* (right) where cell death is distributed evenly between states and cells can transition between states. Observed and simulated values at 72hr are shown to the right of both plots. (**b**) Graph showing the cell state composition of 4 differentiation states vs. DMSO, as in *Figure 3a*, following 72hr of 1µM Trametinib or 1µM BEZ235 treatment then drug washout, examining cell state composition every 3-4 days for 17 days, n=4 with SD. (**c**) Dose response curves showing sensitivity to BEZ235 in untreated cells, or cells treated with Trametinib or BEZ235 then recovered from drug washout for 30 days. ns = not significant, full dose response compared using two-way ANOVA, examining 'interaction'. (**d**) Same as in ***c*** examining sensitivity to Trametinib in untreated or inhibitor-recovered cells.

Supplementary Figure 9 | Targeting DTP states using VIPER informed pathway analysis

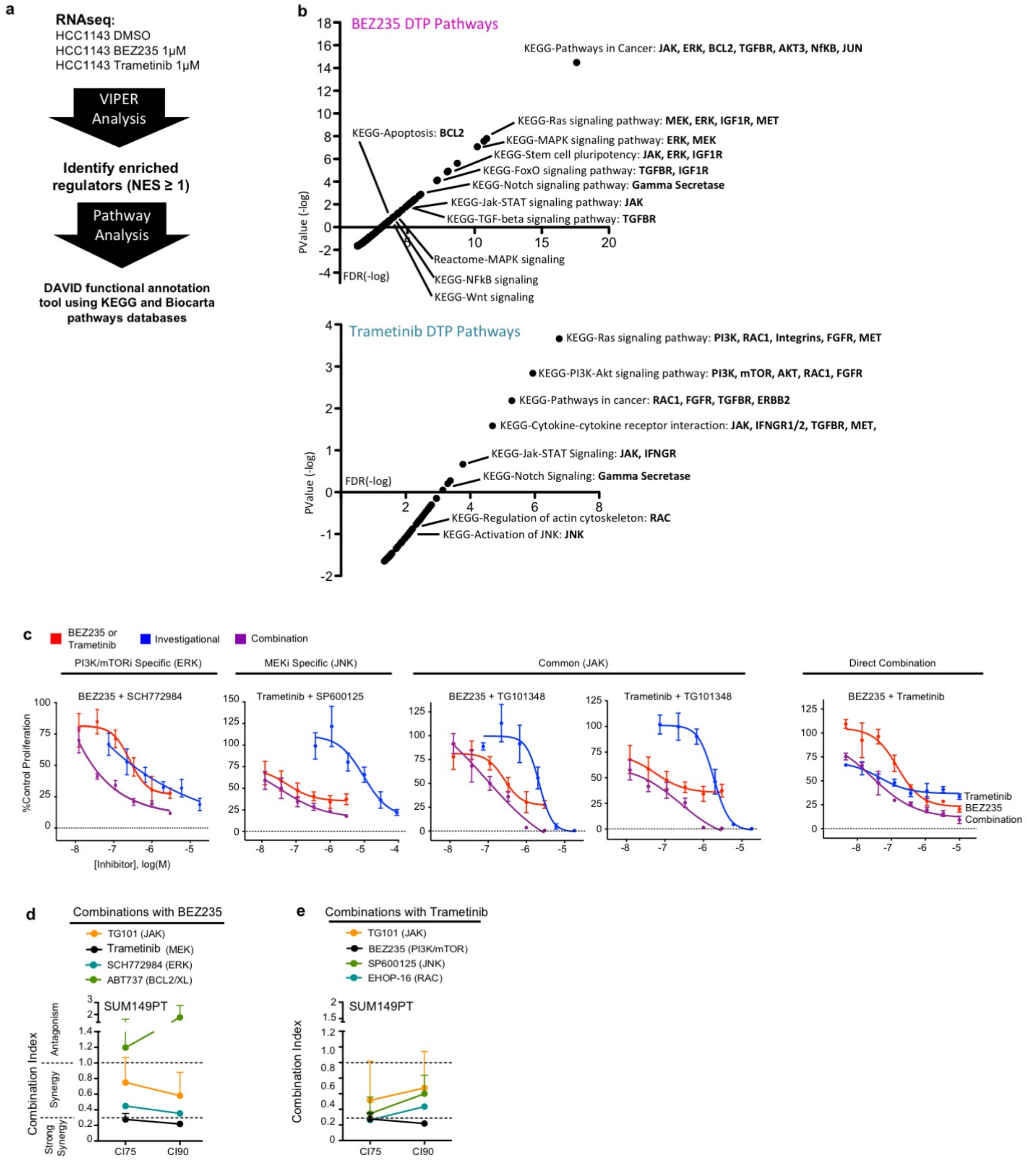

Supplementary Figure 9. Targeting DTP states using VIPER informed pathway analysis.
(**a**) Schematic showing how RNAseq information is analyzed to identify targetable pathway regulators using VIPER and DAVID pathway ontology using the KEGG and Biocarta databases. (**b**) Plots show enriched pathways identified by the DAVID pathway annotation tool, using upregulated VIPER regulators in BEZ235 and Trametinib DTPs as input. P-value (-log) is plotted against FDR values (-log). Select pathways are labeled, and druggable targets within those pathways are listed in bold. (**c**) Dose-response curves examining the viability of HCC1143 cells following combination treatments with BEZ235, Trametinib, and the investigational agents targeting resistant cell-state pathways used in Fig. 4b, n=3 with SD. (**d**) Graph showing combination indices for the agents identified in ***d*** when combined with BEZ235 in SUM149PT cells. (**e**) Graph showing combination indices for the agents identified in ***d*** when combined with Trametinib in SUM149PT cells.

Supplementary Figure 10 | Combination therapy leaves distinct drug-persisting cells that are broadly drug-tolerant and can proliferate and recapitulate heterogeneity upon drug removal.

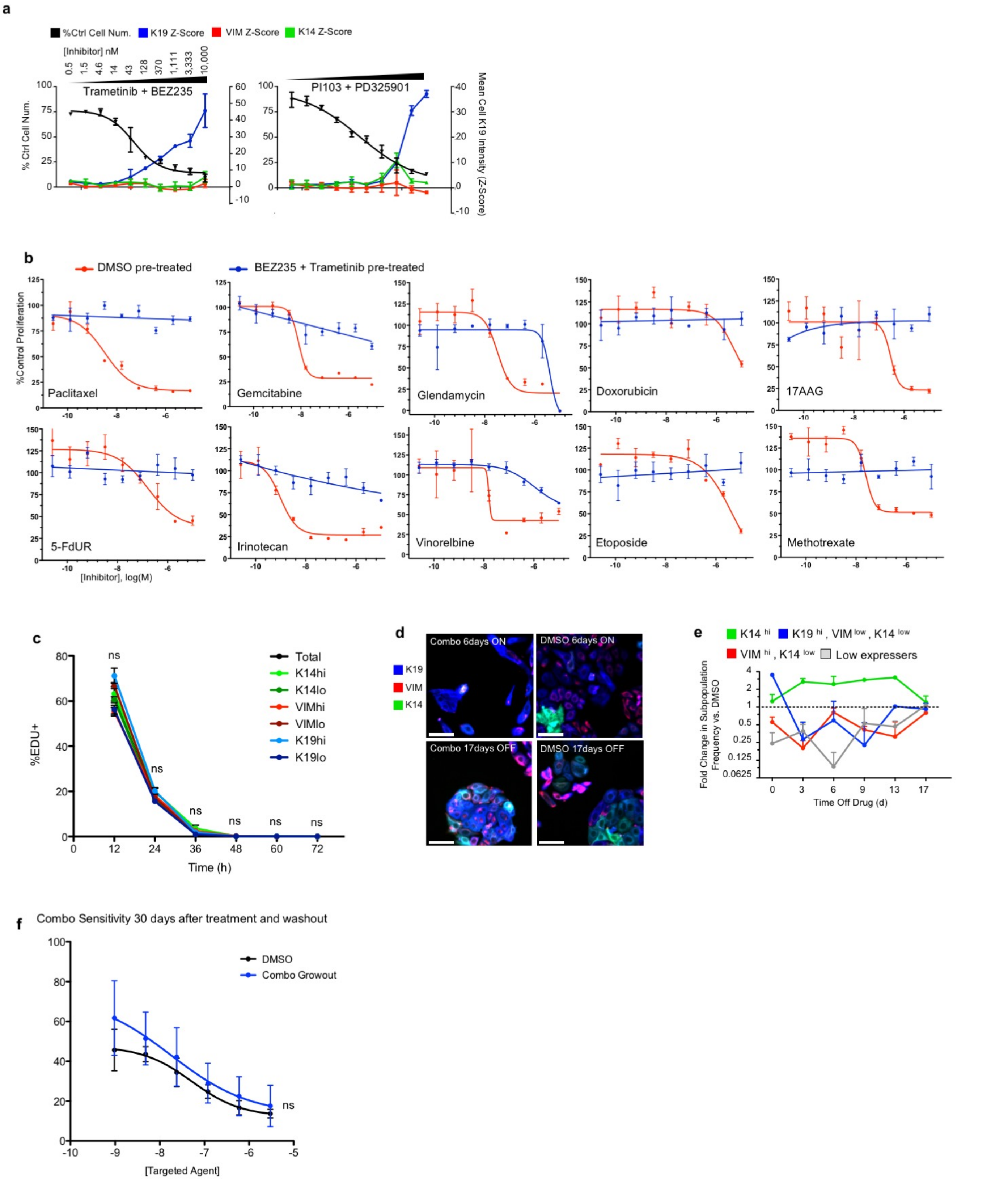

Supplementary Figure 10. Combination therapy leaves distinct drug-persisting cells that are broadly drug-tolerant and can proliferate and recapitulate heterogeneity upon drug removal.
(**a**) Graphs showing Trametinib + BEZ235 treatment-induced changes in cell number (black line, left axis) and population mean-cell intensities (Z-scores, right axis) of K19 (blue), VIM (red), and K14 (green) in HCC1143 following 72hr incubation with increasing doses of two different PI3K/mTORi + MEKi combinations, n=3 with SD. (**b**) Graphs showing the sensitivity of HCC1143 cells to numerous cytotoxic agents following 72h pre-treatment of DMSO, or 72h pre-treatment with 1µM BEZ235 + 1µM Trametinib, followed by 72h incubation with the cytotoxic agent. (**c**) Graph showing the frequency of EdU positive cells that also express high or low levels of K14, K19, or VIM in HCC1143 cells following 72hr treatment with 1µM Trametinib + 1µM BEZ235. ns = not significant, n=2, SD on technical replicates shown. (**d**) Immunofluorescent images of HCC1143 cells treated with 1µM Trametinib + 1µM BEZ235, or DMSO, for 6 days, and then following 17 days of culture after drug washout. (**e**) Graph showing cell state composition of 4 differentiation states as fold change vs. DMSO, as in *Figure 3a, b*, following 72hr of 1µM Trametinib + BEZ235 treatment then drug washout, examining cell state composition every 3-4 days for 17 days, n=4 with SD. (**f**) Dose response curves showing sensitivity to BEZ235 + Trametinib combination treatment in untreated cells, or cells treated with combination for 72hr then recovered from drug washout for 30 days. ns = not significant, full dose response compared using two-way ANOVA, examining ‘interaction’.

Supplementary Figure 11 | JQ1 is not synergistic with BEZ235 in Luminal B lines but shows synergy with Trametinib and Trametinib + BEZ235 combinations in Basal-like lines.

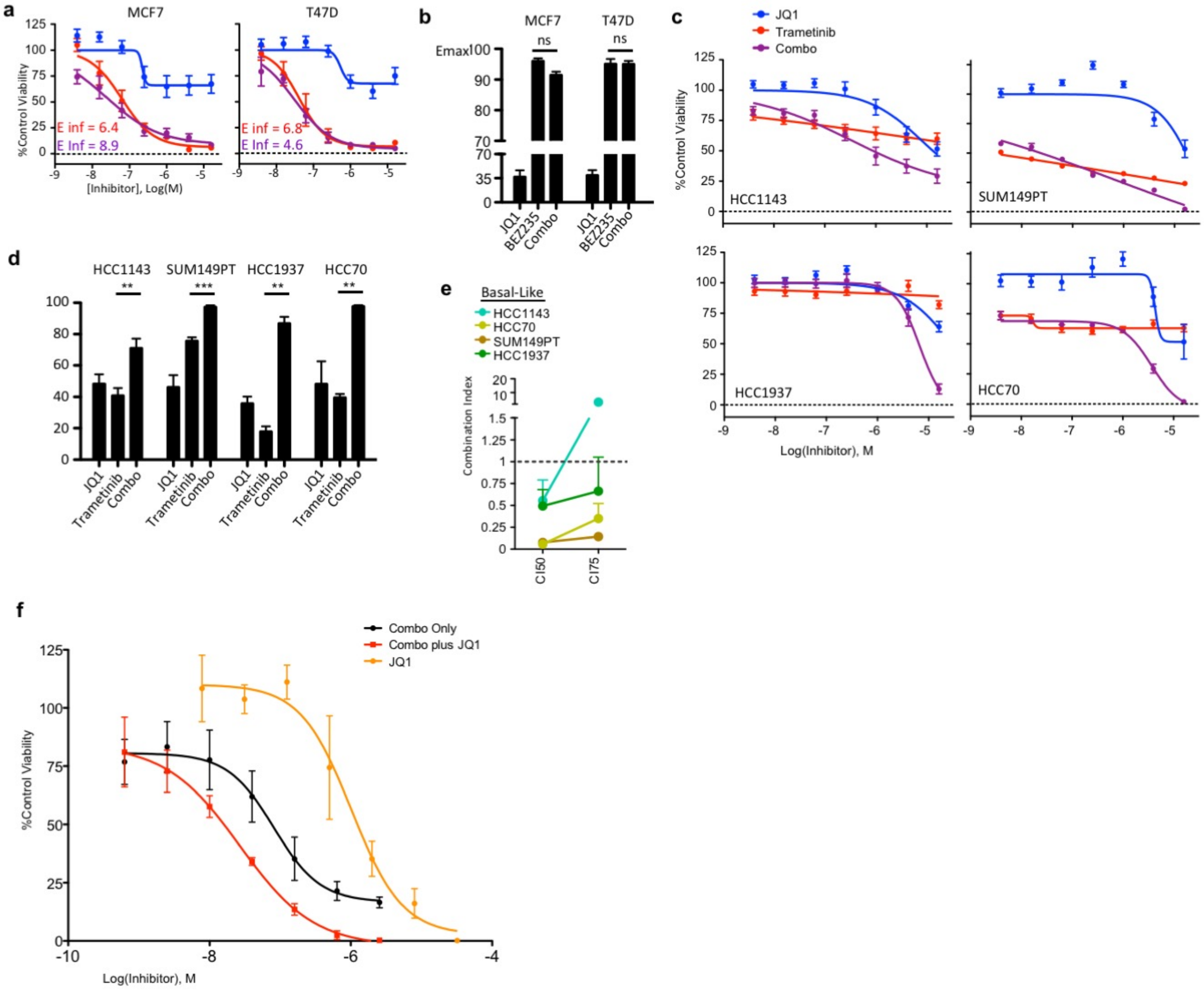

Supplementary Figure 11. JQ1 is not synergistic with BEZ235 in Luminal B lines but shows synergy with Trametinib and Trametinib + BEZ235 combinations in Basal-like lines.

(**a**) Dose-response curves show the efficacy of BEZ235 alone (red), JQ1 alone (blue), or a combination of the two agents (purple, equimolar ratio) in two luminal B cell lines using a colorimetric proliferation assay. E-infinity (Einf) values of single agent BEZ235 (red) and BEZ235 + JQ1 (purple) are displayed. n=4 with SEM. (**b**) Maximal inhibition (Emax) by the agents shown in ***a*** is displayed for MCF7 and T47D, no significant (ns) differences were seen between Emax from BEZ235 and JQ1+BEZ235. (**c**) Dose-response curves of Trametinib, JQ1, and Trametinib + JQ1 in four basal-like cell lines. n=4 with SEM (**c**) Maximal inhibition (Emax) by the agents shown in ***c*** is displayed for each basal like cell line, asterisks depict significant gains in Emax with JQ1 + Trametinib compared to Trametinib alone. (**d**) Maximal inhibition (Emax) by the agents shown in ***c*** is displayed for each basal like cell line, asterisks depict significant gains in Emax with JQ1 + Trametinib compared to BEZ235 alone, *P < 0.05, **P < 0.01, ***P < 0.001, ****P < 0.001. Graph showing combination indices for the BEZ235 + JQ1 drug combination at 75% (CI75), and 90% (CI90) inhibitory doses for 4 basal-like and 2 luminal B BCCLs, n=5 with SEM. (**e**) Graph showing the combination indices of Trametinib + JQ1 at 75% inhibitory values (CI75) and 90% inhibitory values (CI90) for four basal-like cell lines, n=4 with SEM. (**f**) A representative dose response curve of BEZ235 + Trametinib, JQ1 alone, and the three drugs in combination in HCC1143 cells.

Supplementary Figure 12 | Drug treatments enrich open chromatin architecture states observed in subpopulations of DMSO cells.

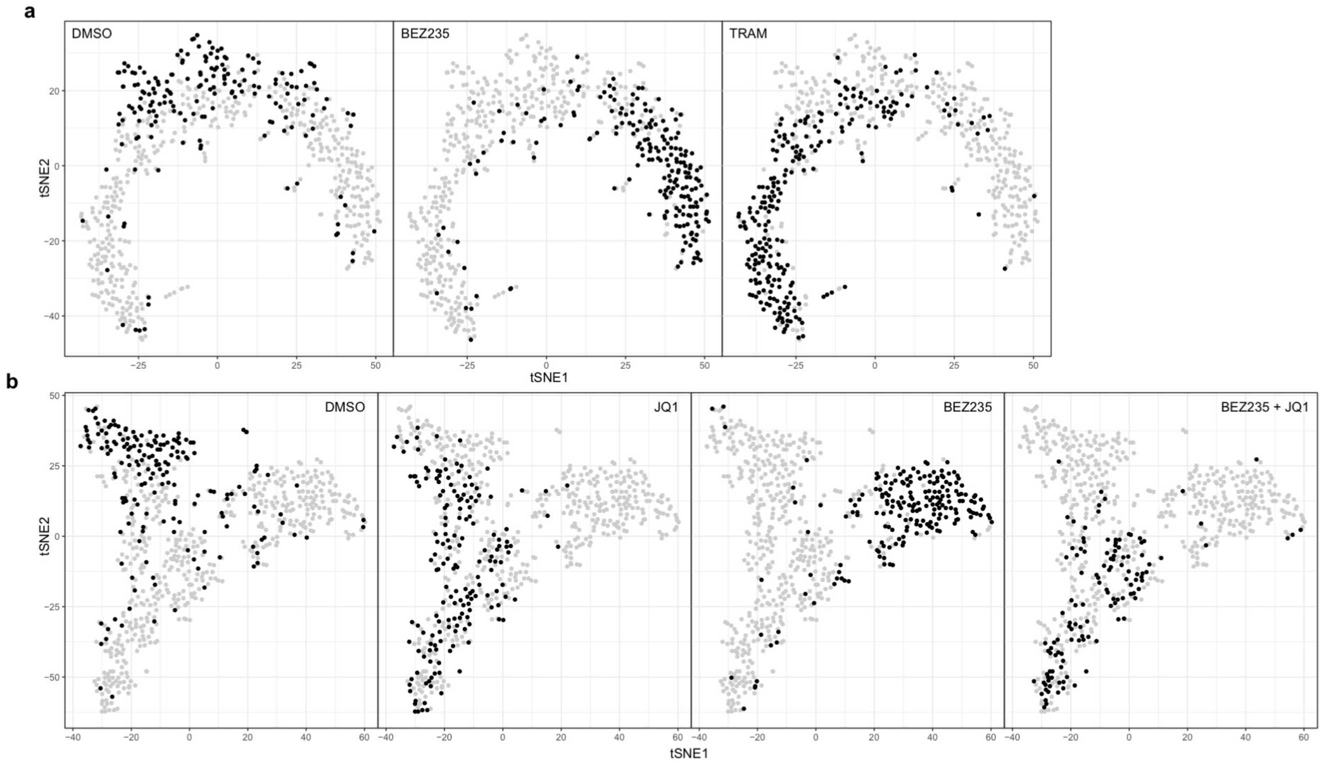

Supplementary Figure 12. Drug treatments enrich open chromatin architecture states observed in subpopulations of DMSO cells.
(**a**) t-SNE plots showing sciATACseq results, highlighting the distribution of DMSO, BEZ235, or Trametinib treated cells in black in three separate plots, all other cells are colored in grey. (**b**) t-SNE plots showing sciATACseq results, highlighting the distribution of DMSO, BEZ235, JQ1, or BEZ235 + JQ1 treated cells in black in four different plots, all other cells are colored in grey.

Supplementary Figure 13 | JQ1 prevents BEZ235-driven open chromatin architecture changes and TF accessibility increases

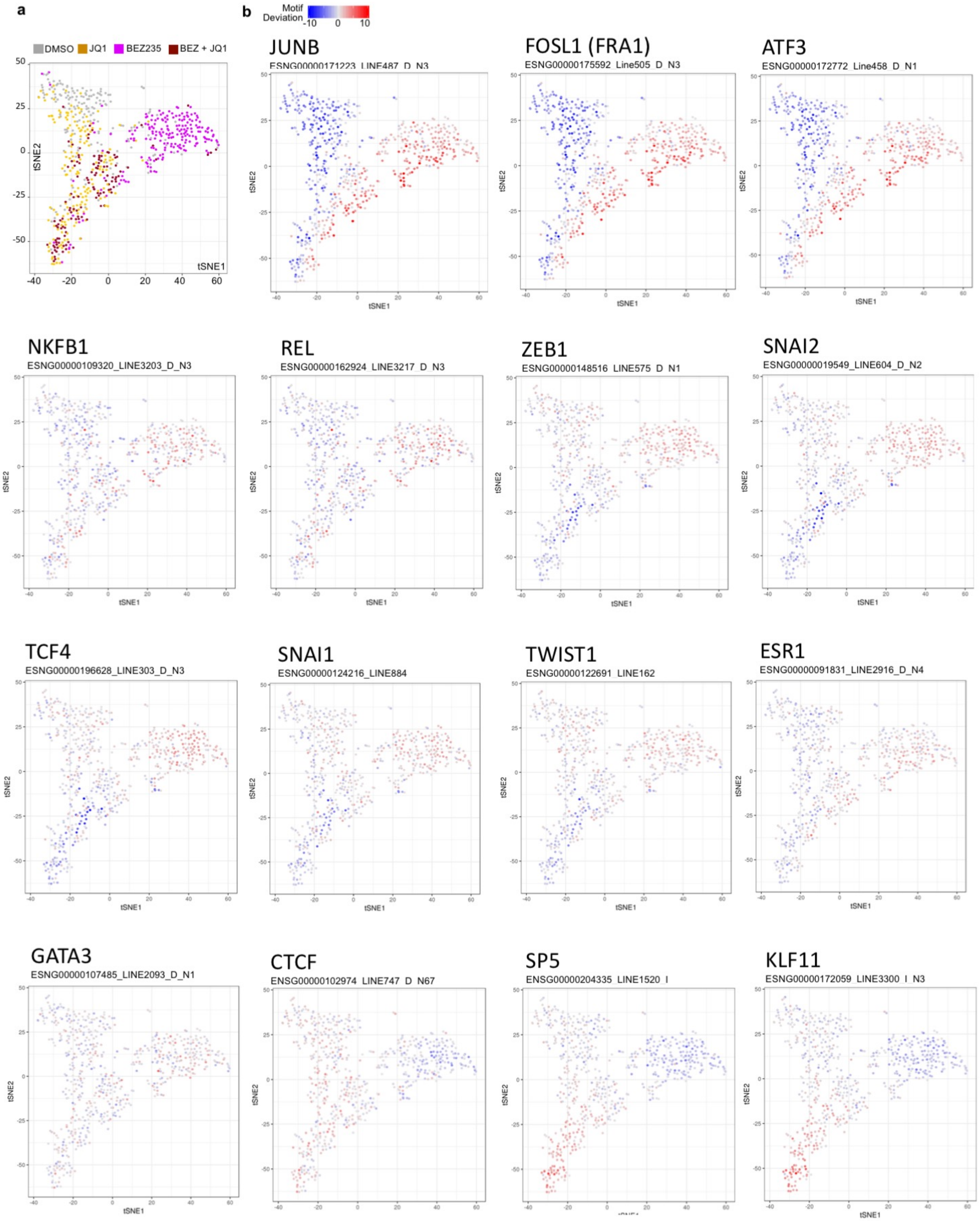

Supplementary Figure 13. JQ1 prevents BEZ235-driven changes to open chromatin architecture and TF motif accessibility.

(**a**) Figure 6c is presented again for reference for the following plots. (**b**) t-SNE plot showing sciATACseq results of HCC1143 cells treated with BEZ235, JQ1, BEZ235 + JQ1 or a DMSO control. Each single cell point is colored based on the level of DNA-binding motif enrichment of the listed transcription factor.

Supplementary Figure 14 | JQ1 prevents open chromatin architecture changes that support BEZ235 DTP-state GATA3 increases.

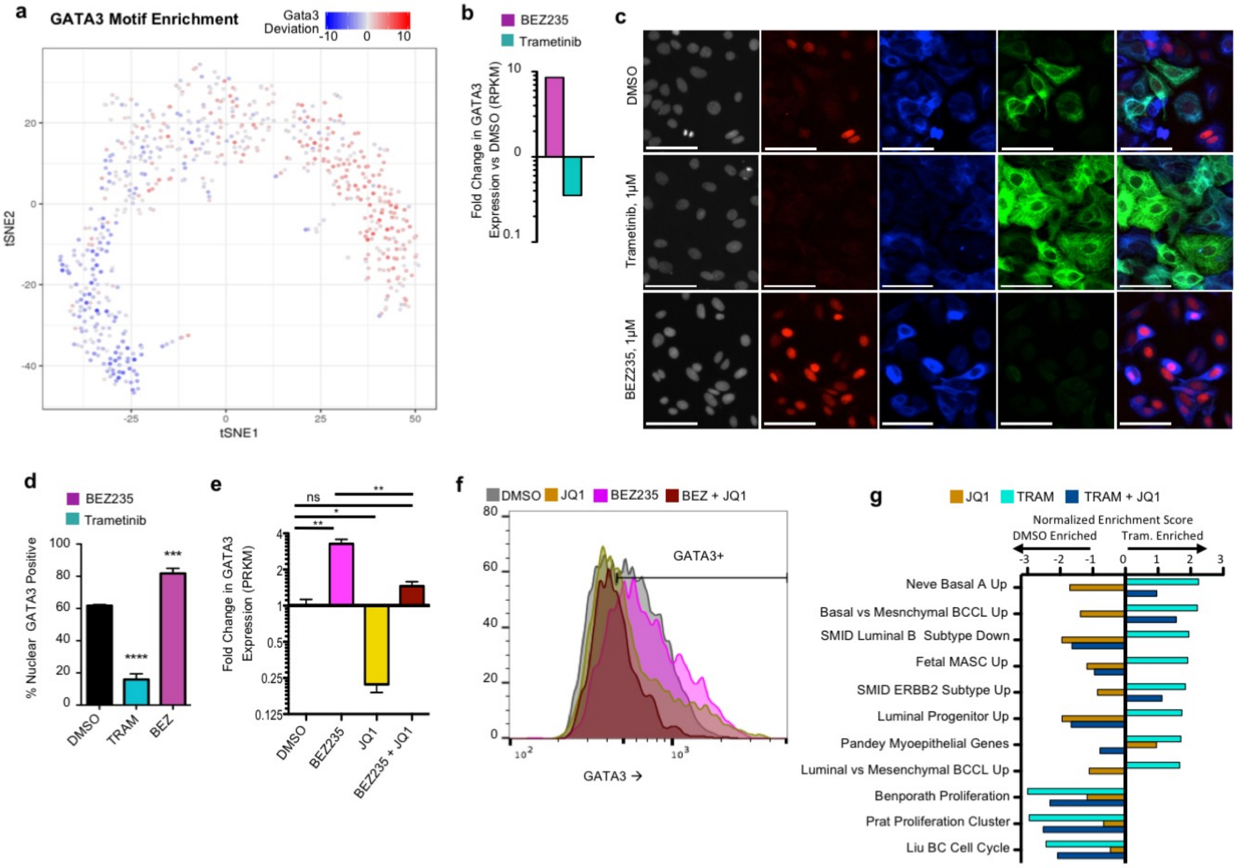

Supplementary Figure 14. JQ1 prevents open chromatin architecture changes that support BEZ235-DTP motif accessibility.

(**a**) GATA3 motif enrichment in the open chromatin regions of DMSO, Trametinib, or BEZ235 treated HCC1143 cells is shown on a t-SNE plot of single cells using sciATACseq. The treatment for each individual cell is displayed by color in *Fig. 6a*, red indicates increased motif enrichment, and blue indicates reduced motif enrichment. (**b**) Graph showing changes in GATA3 gene expression as a fold change vs. DMSO following 6d of 1μM BEZ235 or 1μM Trametinib treatment. (**c**) IF images showing single cell expression of GATA3 (red), K19 (blue) and K14 (green) following 72 of 1μM BEZ235, 1μM Trametinib, or a DMSO control. Scale bars = 100μm. (**d**) Graph showing the quantitation of the frequency of GATA3+ cells in HCC1143 following 72 of 1μM BEZ235, 1μM Trametinib, or a DMSO control. (**e**) A dot plot showing DNA-binding protein motif enrichment analysis between BEZ235 and BEZ235 + JQ1, both normalized to DMSO motif enrichment levels. (**f**) Histograms showing mean-nuclei intensities of GATA3 in HCC1143 cells following 72hr treatment with DMSO (grey), 2μM JQ1 (yellow), 1μM BEZ235 (pink), or BEZ235 + JQ1 (maroon), the gate for GATA3 positivity is shown. (**g**) Graph of GSEA results showing the NES of breast phenotype genesets shown to be significantly enriched ($p < 0.05$) following 72hr of Trametinib treatment, with the subsequent NES of that geneset in JQ1-treated, and Trametinib + JQ1-treated cells shown adjacently.

Supplementary Figure 15 | JQ1 combines with BEZ235 *in vivo* to reduce tumor volume and suppress cell-state transitions.

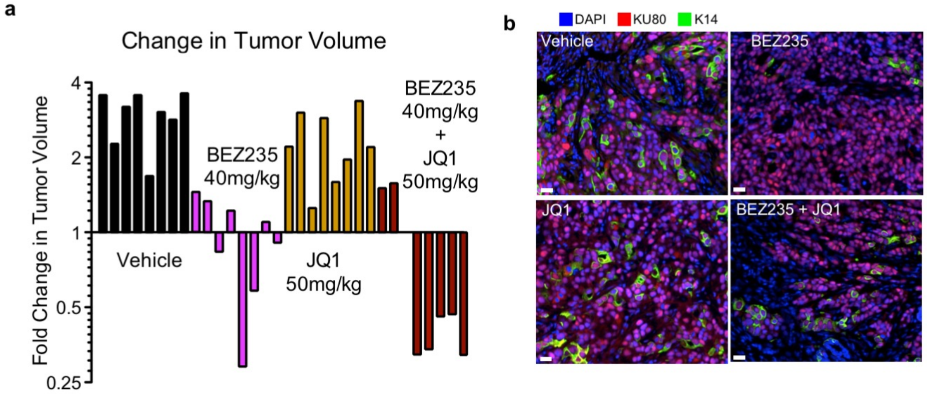

Supplementary Figure 15. JQ1 combines with BEZ235 *in vivo* to reduce tumor volume and suppress cell-state transitions.

(**a**) A waterfall plot shows the change in individual tumor volume, comparing treatment initiation size to size at resection (treatment day 21). Bars are colored by treatment: Vehicle (black), BEZ235 (magenta), JQ1 (yellow), BEZ235 + JQ1 (maroon). (**b**) Representative IF images of HCC70 xenograft tumors treated with vehicle, BEZ235, JQ1, or the combination of JQ1 + BEZ235 showing DAPI (blue), human specific marker Ku80 (red) and K14 (green), scale bars = 100µm.

Supplementary Table 1 | Variants and indels identified in whole exome sequencing analysis comparing treated HCC1143 cells.

VAF = Variant allele frequency, IGR = Intergenic regions, MQ = Mapping quality, RNA = variant/indel found in shared sequence of non-coding RNA

Comparison	Contig	Position	Treatment Reference Count	Treatment Alt Count	Tumor VAF	Gene	Variant Classification	Variant Type	Genomic Change (Ref>Alt Allele)	Protein Change	Technical Concerns
BEZ235 vs DMSO	10	82182414	23	7	23	FAM213A	Intron	SNV	G>T	N/A	Error prone region, Near multiple indels, Low depth
BEZ235 vs DMSO	15	81642092	30	7	19	TMC3	Intron	SNV	A>T	N/A	Error prone region, Near multiple indels, T-rich/T-repetitive, Low depth
BEZ235 vs DMSO	19	39422827	36	7	16	MRPS12	Intron	SNV	G>A	N/A	Error-prone region (A-rich/A-repetitive)
Trametinib vs DMSO	2	171094874	27	5	16	MYO3B	Intron	DEL	TTC>T	N/A	Repetitive TC Region
Trametinib vs DMSO	15	90340666	27	3	10	ANPEP	Intron	DEL	TT>T	N/A	Repetitive T Region
Trametinib vs DMSO	19	15692663	36	4	10	Unknown	IGR	SNV	C>G	N/A	
Trametinib vs DMSO	22	38903371	31	5	14	DDX17	Intron	DEL	AA>A	N/A	Repetitive A Region
Trametinib vs DMSO	X	76632551	27	3	10	Unknown	IGR	INS	A>AA	N/A	Repetitive A Region
BEZ235 vs Trametinib	1	12023765	36	4	10	PLOD1	Intron	DEL	TCCTCC>TCC	N/A	Repetitive TCC Region
BEZ235 vs Trametinib	3	70175377	63	9	13	Unknown	IGR	SNV	C>T	N/A	T-variant rich region, Present in DMSO, low VAF
BEZ235 vs Trametinib	3	110866077	27	6	18	PVRL3	Intron	SNV	C>G	N/A	Low depth in Tram, Present in DMSO
BEZ235 vs Trametinib	7	116772155	39	5	11	ST7	Intron	INS	T>TT	N/A	Repetitive T Region
BEZ235 vs Trametinib	10	18122510	27	6	18	MRC1	Intron	SNV	G>A	N/A	Low MQ region, Error-prone region (A-rich/A-repetitive), Present in DMSO, Low depth
BEZ235 vs Trametinib	10	120467206	39	7	15	CACUL1	Intron	SNV	C>A	N/A	Error-prone region (indel-rich, A-rich/A-repetitive), Present in DMSO
BEZ235 vs Trametinib	16	46402854	27	3	10	Unknown	IGR	SNV	A>T	N/A	Low MQ region, Low MQ reads, Low VAF, Low depth
BEZ235 vs Trametinib	19	39422827	36	7	16	MRPS12	Intron	SNV	G>A	N/A	Error-prone region (A-rich/A-repetitive)
BEZ235 vs Trametinib	19	53874105	27	4	13	ZNF525	Intron	SNV	G>A	N/A	Present in DMSO, Low VAF, Low depth
Trametinib vs BEZ235	1	148944673	44	5	10	RP11-14N7.2	lincRNA	SNV	A>G	N/A	Low VAF, Low MQ region, Present in BEZ235
Trametinib vs BEZ235	1	151184288	30	4	12	PIP5K1A	Intron	INS	A>AA	N/A	Repetitive A Region
Trametinib vs BEZ235	3	73254936	34	5	13	Unknown	IGR	DEL	ACAC>A	N/A	Repetitive AAC/ACC Region
Trametinib vs BEZ235	5	148686810	29	4	12	AFAP1L1	Intron	DEL	AA>A	N/A	Repetitive A Region
Trametinib vs BEZ235	7	61845544	26	4	13	Unknown	IGR	SNV	A>T	N/A	Low MQ region, Present in DMSO, indel/GA-rich/GA-repetitive, Low tumor VAF, Low depth
Trametinib vs BEZ235	9	8858093	29	4	12	PTPRD	Intron	DEL	CCTCCT>CCT	N/A	Repetitive CCT Region
Trametinib vs BEZ235	10	82007541	41	6	13	Unknown	IGR	SNV	C>T	N/A	Low MQ region, GCC-repetitive, Multiple proximal indels, Low tumor VAF, Present in DMSO
Trametinib vs BEZ235	20	23066788	60	8	12	CD93	Frame_Shift_Del	DEL	AGCAGC>AGC	ENSP00000246006:p.Leu15fs	Also present in DMSO; CAG/Leucine repetitive regions
Trametinib vs BEZ235	21	10139739	35	4	10	Unknown	IGR	SNV	C>A	N/A	Low MQ region, Low tumor VAF, Present in DMSO
Trametinib vs BEZ235	21	18882608	28	4	13	Unknown	IGR	SNV	T>C	N/A	Low tumor VAF, Low depth, Present in DMSO
Trametinib vs BEZ235	22	32084062	78	9	10	PRR14L	Intron	SNV	C>A	N/A	Low tumor VAF, Error-prone region
DMSO vs Trametinib	1	185088963	31	4	11	TRMT1L	3'UTR	DEL	AA>A	N/A	Repetitive A Region
DMSO vs Trametinib	5	150916828	34	4	11	FAT2	Intron	DEL	TGGTGG>TGG	N/A	Repetitive TGG Region
DMSO vs Trametinib	9	123715210	27	3	10	C5	Intron	DEL	TT>T	N/A	Repetitive T Region
DMSO vs Trametinib	10	987247	27	3	10	RP11-363N22.2	RNA	DEL	TTTTG>T	N/A	Repetitive TTTG Region
DMSO vs Trametinib	18	20889493	41	5	11	TMEM241	Intron	SNV	A>T	N/A	Low tumor VAF, Variant rich region
DMSO vs BEZ235	1	150583473	27	3	10	ENSA	Intron	SNV	A>T	N/A	Low tumor VAF, Low depth, Error-prone region
DMSO vs BEZ235	1	229613232	73	13	15	NUP133	Intron	SNV	G>A	N/A	Low tumor VAF, Error-prone region, Error-prone site, AT-rich/AT-repetitive
DMSO vs BEZ235	7	143183523	36	4	10	EPHA1-AS1	RNA	DEL	CC>C	N/A	
DMSO vs BEZ235	8	105450965	33	5	13	DPYS	Intron	DEL	GAG>G	N/A	Repetitive AG Region
DMSO vs BEZ235	10	987247	27	3	10	RP11-363N22.2	RNA	DEL	TTTTG>T	N/A	Repetitive TTTG Region
DMSO vs BEZ235	10	32833354	36	5	12	CCDC7	Intron	DEL	CTG>C	N/A	Repetitive TG Region
DMSO vs BEZ235	10	45634788	76	11	13	Unknown	IGR	SNV	G>T	N/A	Error-prone region, A-repetitive, Low tumor VAF
DMSO vs BEZ235	12	73210078	32	4	11	Unknown	IGR	SNV	C>T	N/A	Low tumor VAF, Low MQ site and region, Poly-N repeats
DMSO vs BEZ235	18	20889493	41	5	11	TMEM241	Intron	SNV	A>T	N/A	Low tumor VAF, Low depth, Possible error-prone region
DMSO vs BEZ235	19	9415630	25	6	19	ZNF699	Intron	SNV	T>G	N/A	Low tumor VAF, Variant rich region, A-repetitive, low depth
DMSO vs BEZ235	21	11081322	23	8	26	BAGE2	RNA	SNV	G>A	N/A	Variant rich region, low depth in BEZ235
DMSO vs BEZ235	22	21066700	54	6	10	PI4KA	Intron	SNV	G>A	N/A	Low tumor VAF

Supplementary Table 1. Variants and indels identified in whole exome sequencing analysis comparing treated HCC1143 cells.

A summary of variants and indels identified by MuTect analysis between treatment conditions in HCC1143 whole exome sequencing data. For each variant and indel identified in a given pairwise comparison, the contig, genomic position, count of the reference allele, count of the variant allele, tumor variant allele frequency (VAF), gene name, location classification (e.g. exon, intron, intergenic region), variant type (e.g. single nucleotide variant, SNV; insertion, INS; deletion, DEL), genomic change, protein change, and technical concerns for the call are provided.

Supplementary Table 2 | Variants and indels identified in whole exome sequencing analysis of treated HCC1143 cells compared to a germline control.

VAF = Variant allele frequency, IGR = Intergenic regions, RNA = variant/indel found in shared sequence of non-coding RNA

Chromosome	Position	Reference Allele	Alternate Allele	VAF in DMSO	VAF in BEZ235	VAF in Trametinib	Gene	Variant Classification	Variant Type	Genomic Change	Protein Change	Commonality
4	70079871	A	T	100	100	100	UGT2B11	Silent	SNV	4:g.70079871A>T	None	Present in all three treatments
6	33382010	GT	G	53	51	49	PHF1	Intron	DEL	6:g.33382010delGT	N/A	
7	143982758	C	A	9	11	6	ARHGEF35	Intron	SNV	7:g.143982758C>A	N/A	
10	42534628	ATT	A	22	19	26	Unknown	IGR	DEL	10:g.42534628delATT	N/A	
10	51769100	G	T	13	18	15	AGAP6	Missense_Mutation	SNV	10:g.51769100G>T	ENSP00000363168:p.Lys382Asn	
11	49896522	T	C	11	17	19	Unknown	IGR	SNV	11:g.49896522T>C	N/A	
11	49896523	G	A	11	17	19	Unknown	IGR	SNV	11:g.49896523G>A	N/A	
11	55059652	C	T	41	35	37	Unknown	IGR	SNV	11:g.55059652C>T	N/A	
11	55890060	A	G	44	37	39	OR8H3	Missense_Mutation	SNV	11:g.55890060A>G	ENSP00000323928:p.Asp71Gly	
14	70713380	G	A	8	9	6	ADAM21P1	RNA	SNV	14:g.70713380G>A	N/A	
14	70925214	C	T	20	20	15	ADAM21	Missense_Mutation	SNV	14:g.70925214C>T	ENSP00000474385:p.Ser333Phe	
16	84529249	ACTG	A	35	37	34	TLDC1	Intron	DEL	16:g.84529249delACTG	N/A	
19	55258778	G	A	60	63	63	KIR2DL3	Intron	SNV	19:g.55258778G>A	N/A	
15	74365059	C	T	13	N/A	10	GOLGA6A	Intron	SNV	15:g.74365059C>T	N/A	Present in Trametinib and DMSO
2	92317863	T	C	N/A	N/A	9	Unknown	IGR	SNV	2:g.92317863T>C	N/A	Tram Only
4	49637265	T	G	9	N/A	N/A	Unknown	IGR	SNV	4:g.49637265T>G	N/A	DMSO only
X	49360749	C	G	8	N/A	N/A	GAGE2A	Intron	SNV	X:g.49360749C>G	N/A	DMSO only
10	42387802	A	T	N/A	10	N/A	Unknown	IGR	SNV	10:g.42387802A>T	N/A	BEZ235 only
10	42394915	G	T	N/A	9	N/A	Unknown	IGR	SNV	10:g.42394915G>T	N/A	BEZ235 only

Supplementary Table 2. Variants and indels identified in whole exome sequencing analysis of treated HCC1143 cells compared to a germline control.

A summary of variants and indels identified by MuTect2 analysis between whole exome data from Trametinib, BEZ235, or DMSO-treated HCC1143 cells and published whole exome data of a patient-matched normal cell line, HCC1143-BL. For each variant and indel identified, the chromosome, position, reference allele, alternate allele, variant allele frequency (VAF) in DMSO, VAF in BEZ235, VAF in Trametinib, gene name, variant classification, variant type, genomic change, protein change, and commonality with other treatment groups are provided.

Supplementary Methods

Computational Modeling

The purpose of this document is to explain the fold change simulations in greater detail (Fig. 3h, Supplementary Fig. 8a). Motivation, notation, and the dynamical system representation are discussed. Details on model identification and fold change calculations follow.

I. Motivation

We used mathematical modeling to examine if Darwinian selection or cell-state transition could explain the differentiation-state trends observed in the HCC1143 cell line treated with Trametinib or BEZ235. Our models were identified with experimental data and served as tools to investigate the feasibility of these biological hypotheses.

Recall from Fig. 3a that the proportion of $K14^{high}$ cells increased while the proportion of $K14^{low}$ cells decreased over time following treatment with Trametinib (vs. DMSO); treatment with BEZ235 induced the reverse trend to a lesser degree. We asked which population-level phenomena might explain the observed outcomes: asymmetrical cell-state death, cell-state transition, or asymmetrical cell-state division. The last option is unlikely based on EdU+ analysis (Fig. 3b). To examine the remaining options, we tested the following hypotheses via computational modeling:

- *Darwinian selection*. Asymmetrical cell-state death can explain the observations.
- *Cell-state transition*. Cell-state transition can explain the observations under evenly distributed cell-state death.

Under the Darwinian selection hypothesis, death was allocated to favor the observed experimental outcome (Trametinib vs. DMSO: all death in $K14^{low}$; BEZ235 vs. DMSO: all death in $K14^{high}$). However, even under these favorable assumptions, fold change simulations could not match the empirical observations (Fig. 3h (left), Supplementary Fig. 8a (left)).

Under the cell-state transition hypothesis, however, death was allocated between cell-states in an unbiased fashion, a realistic guess for the true death distribution. Interestingly, in this more natural setting, it was found that simulations with cell-state transition enabled could match the experimental outcome (Fig. 3h (right), Supplementary Fig. 8a (right)).

While model-based conclusions do not constitute truth, these findings do suggest that cell-state transition plays a more important role than state-selective death in driving drug-induced differentiation-state enrichment following treatment with certain targeted therapies.

II. Notation

Symbols and definitions are below. All terms are agent-specific (e.g., DMSO, Trametinib). *Gain* is the discrete-time analog of *rate*; the latter is used in the main text because it is expected to be more familiar to most readers. Terms labeled ρ are parameters of the system dynamics[1].

Symbol	Definition
$\#K14^{lo}_{obs}$	observed number of $K14^{low}$ cells via image cytometry
$\#K14^{hi}_{obs}$	observed number of $K14^{high}$ cells via image cytometry
f^{dead}_{obs}	observed fraction of dead cells via YO-PRO-1
$\#K14^{lo}_{live,\,tr}$	number of $K14^{low}$ live cells for model training
$\#K14^{hi}_{live,\,tr}$	number of $K14^{high}$ live cells for model training
$\#dead_{tr}$	number of dead cells for model training

ρ_{hD}	death gain, K14^{high}
ρ_{lD}	death gain, K14^{low}
$\rho_{h\to l}$	transition gain from K14^{high} to K14^{low}
$\rho_{l\to h}$	transition gain from K14^{low} to K14^{high}
ρ_h	cell division gain, K14^{high}
ρ_l	cell division gain, K14^{low}
$\#K14^{lo}_{live,\ sim}$	number of K14^{low} live cells generated via dynamics simulation
$\#K14^{hi}_{live,\ sim}$	number of K14^{high} live cells generated via dynamics simulation
$\#dead_{sim}$	number of dead cells generated via dynamics simulation

III. Dynamical system

A linear time-invariant dynamical system was chosen to model cell-state interactions, $x[k+1] = A \cdot x[k]$, where state vector x = (number of K14^{high} live cells, number of K14^{low} live cells, number of dead cells)$^T \in \mathbb{R}^3$, [k, k+1) is a 12-hour interval, and dynamics matrix $A \in \mathbb{R}^{3x3}$ is parameterized as follows:

$$A = \begin{bmatrix} \rho_h - \rho_{h\to l} - \rho_{hD} & \rho_{l\to h} & 0 \\ \rho_{h\to l} & \rho_l - \rho_{l\to h} - \rho_{lD} & 0 \\ \rho_{hD} & \rho_{lD} & 1 \end{bmatrix}.$$

Values of the dynamics parameters were identified using training data computed from timecourse data in a manner specified by hypothesis and chemical agent (Sec. III). Thus, there is a *unique* dynamics matrix for each (hypothesis, agent) pair:

**Darwinian selection hypothesis**	**Cell-state transition hypothesis**
$A_{DMSO} = \begin{bmatrix} 1.307 & 0 & 0 \\ 0 & 1.307 & 0 \\ 0.006 & 0.006 & 1 \end{bmatrix}$	$A_{DMSO} = \begin{bmatrix} 0.301 & 0.337 & 0 \\ 1 & 1.014 & 0 \\ 0.049 & 0 & 1 \end{bmatrix}$
$A_{Trametinib} = \begin{bmatrix} 1.117 & 0 & 0 \\ 0 & 0.946 & 0 \\ 0 & 0.171 & 1 \end{bmatrix}$	$A_{Trametinib} = \begin{bmatrix} 1.023 & 0.241 & 0 \\ 0.014 & 0.832 & 0 \\ 0.036 & 0 & 1 \end{bmatrix}$
$A_{BEZ235} = \begin{bmatrix} 0.900 & 0 & 0 \\ 0 & 1.132 & 0 \\ 0.232 & 0 & 1 \end{bmatrix}$	$A_{BEZ235} = \begin{bmatrix} 0.880 & 0 & 0 \\ 0.149 & 1.096 & 0 \\ 0.068 & 0 & 1 \end{bmatrix}$

Propagating a given matrix *N* time steps forward from initial condition, $x_0 \in \mathbb{R}^3$, yields state vector at time *N*, $x[N] = A^N \cdot x_0$. For each (hypothesis, agent) pair, quantities of K14^{high} live cells, K14^{low} live cells, and dead cells were generated over time in this way using the appropriate dynamics matrix.

IV. On training data and parameter constraints for model identification

Dynamical models were generated from timecourse data to examine the feasibility of two distinct hypotheses (Darwinian selection, Cell-state transition). The tables below summarize training data computations and parameter constraints for such models under each hypothesis in the context of Trametinib vs. DMSO. (For BEZ235 vs. DMSO, swap K14^{high} and K14^{low}.) Additional practical constraints (omitted below) were enforced, e.g., nonnegative gains[1]

$K14^{high}$ Darwinian selection hypothesis

Agent	Assumptions on cell-state death & transition	How to compute training data for model identification from timecourse observations	Parameter constraints
Trametinib	All death in $K14^{low}$<br>Transition inhibited	$\#dead_{tr} = f^{dead}_{obs} \cdot (\#K14^{lo}_{obs} + \#K14^{hi}_{obs})$<br>$\#K14^{lo}_{live,\,tr} = \#K14^{lo}_{obs} - \#dead$<br>$\#K14^{hi}_{live,\,tr} = \#K14^{hi}_{obs}$	$\rho_{hD} = 0$<br>$\rho_{h\rightarrow l} = \rho_{l\rightarrow h} = 0$<br>$\rho_h = \rho_l$
DMSO	Equal death $K14^{high}$ & $K14^{low}$<br>Transition inhibited	$\#dead_{tr} = f^{dead}_{obs} \cdot (\#K14^{lo}_{obs} + \#K14^{hi}_{obs})$<br>$\#K14^{lo}_{live,\,tr} = (1 - f^{dead}_{obs}) \cdot \#K14^{lo}_{obs}$<br>$\#K14^{hi}_{live,\,tr} = (1 - f^{dead}_{obs}) \cdot \#K14^{hi}_{obs}$	$\rho_{hD} = \rho_{lD}$;<br>$\rho_{h\rightarrow l} = \rho_{l\rightarrow h} = 0$<br>$\rho_h = \rho_l$

Cell-state transition hypothesis

Agent	Assumptions on cell-state death & transition	How to compute training data for model identification from timecourse observations	Parameter constraints
Trametinib	Equal death $K14^{high}$ & $K14^{low}$<br>Transition enabled	$\#dead_{tr} = f^{dead}_{obs} \cdot (\#K14^{lo}_{obs} + \#K14^{hi}_{obs})$<br>$\#K14^{lo}_{live,\,tr} = (1 - f^{dead}_{obs}) \cdot \#K14^{lo}_{obs}$<br>$\#K14^{hi}_{live,\,tr} = (1 - f^{dead}_{obs}) \cdot \#K14^{hi}_{obs}$	$\rho_h = \rho_l$
DMSO	Equal death $K14^{high}$ & $K14^{low}$<br>Transition enabled	$\#dead_{tr} = f^{dead}_{obs} \cdot (\#K14^{lo}_{obs} + \#K14^{hi}_{obs})$<br>$\#K14^{lo}_{live,\,tr} = (1 - f^{dead}_{obs}) \cdot \#K14^{lo}_{obs}$<br>$\#K14^{hi}_{live,\,tr} = (1 - f^{dead}_{obs}) \cdot \#K14^{hi}_{obs}$	$\rho_h = \rho_l$

V. How to compute simulated fold change

Our computational models provided *simulated* quantities of $K14^{high}$ live cells, $K14^{low}$ live cells, and dead cells. Image cytometry provided *measured* quantities of $K14^{high}$ cells (live & dead) and $K14^{low}$ cells (live & dead); phenotypes of dead cells were unobservable. Thus, the simulated values were processed further to quantify $K14^{high}$ cells (live & dead) and $K14^{low}$ cells (live & dead) for direct comparison with biology (Fig. 3h, Supplementary Fig. 8a). These steps in the context of Trametinib vs. DMSO are below. (For BEZ235 vs. DMSO, swap $K14^{high}$ and $K14^{low}$.) Let agent $i \in$ {Trametinib, DMSO} and phenotype $p \in \{K14^{hi}, K14^{lo}\}$. For each time $k \in$ {0, 12, …, 72} hr, simulated fold change of phenotype p at time k is the ratio, $\frac{[\,fraction^{p}_{sim}\,]_{time\ k,\ Trametinib}}{[\,fraction^{p}_{sim}\,]_{time\ k,\ DMSO}}$, in which simulated phenotype p fraction is defined as:

$$[\,fraction^{K14^{lo}}_{sim}\,]_{time\ k,\ agent\ i} = \left[\frac{\#K14^{lo}_{sim}}{\#K14^{hi}_{live,\ sim} + \#K14^{lo}_{live,\ sim} + \#dead_{sim}}\right]_{time\ k,\ agent\ i} \quad (1a)$$

$$[\,fraction^{K14^{hi}}_{sim}\,]_{time\ k,\ agent\ i} = \left[\frac{\#K14^{hi}_{sim}}{\#K14^{hi}_{live,\ sim} + \#K14^{lo}_{live,\ sim} + \#dead_{sim}}\right]_{time\ k,\ agent\ i}. \quad (1b)$$

The denominator of (1), cell population total, is obtained directly via dynamics simulation. The numerator of (1) is the *in silico* quantity of phenotype p cells (live & dead) at time k after initial treatment with agent i, which requires additional computation based on (hypothesis, agent) pair. Under $K14^{high}$ Darwinian selection hypothesis, death is allocated to the weaker cell state, $K14^{low}$, in entirety under Trametinib:

$$[\,\#K14^{lo}_{sim}\,]_{time\ k,\ Trametinib} = [\,\#K14^{lo}_{live,\ sim} + \#dead_{sim}\,]_{time\ k,\ Trametinib} \quad (2)$$

$$[\,\#K14^{hi}_{sim}\,]_{time\ k,\ Trametinib} = [\,\#K14^{hi}_{live,\ sim}\,]_{time\ k,\ Trametinib} \quad (3)$$

However, under the cell-state transition hypothesis, Trametinib-induced death is evenly distributed between cell states:

$$[\,\#K14^{lo}_{sim}\,]_{time\ k,\ Trametinib} = \left[\frac{\#K14^{lo}_{live,\ sim}}{1 - fraction^{dead}_{sim}}\right]_{time\ k,\ Trametinib} \quad (4)$$

$$\left[\#K14^{hi}_{sim} \right]_{time\ k,\ Trametinib} = \left[\frac{\#K14^{hi}_{live,\ sim}}{1 - fraction^{dead}_{sim}} \right]_{time\ k,\ Trametinib}, \quad (5)$$

such that simulated dead fraction at time *k* under agent *i* is:

$$\left[fraction^{dead}_{sim} \right]_{time\ k,\ agent\ i} = \left[\frac{\#dead_{sim}}{\#K14^{hi}_{live,\ sim} + \#K14^{lo}_{live,\ sim} + \#dead_{sim}} \right]_{time\ k,\ agent\ i}. \quad (6)$$

For both hypotheses, because $K14^{high}$ and $K14^{low}$ should be similarly fit in absence of therapy, death is evenly distributed between cell states under DMSO. Replace Trametinib in (4) and (5) with DMSO, and the computation follows.

Whole exome sequencing

Genomic DNA (2ug) derived from tissue-cultured HCC1143 cells treated with DMSO, 1uM BEZ235, or 1uM Trametinib was sonicated using a Covaris E220 Focused Ultrasonicator (Covaris, Inc.) to an average size of 150bp. Libraries were prepared in triplicate using 500ng of above fragmented gDNA with the KAPA HyperPrep Library Preparation Kit (KAPA Biosystems) using Agilent SureSelect XT Target Enrichment System Reagents (Agilent) and custom adaptors containing 14bp degenerate UMI and 8bp fixed sample-indices. Whole-exome hybridization capture was carried out using Agilent Human All Exome V5 Kit, following manufacturer's protocols. Samples were multiplexed and 100bp, paired-end sequenced following cluster generation on the Illumina HiSeq 2500 by the OHSU Massively Parallel Sequencing Shared Resource. Raw paired-end sequencing reads (100bp) output in FastQ format by the HiSeq 2500 were aligned using BWA MEM (0.7.12) software to the full hg19 genomic assembly (GATK, Broad Institute). Picard tools (1.119), SAMtools[2], and GATK (3.3-0) were used to sort, index, remove PCR duplicates, locally realign and merge bam files, as well as to generate target coverage and duplication metrics (http://broadinstitute.github.io/picard). After data processing, a mean of 300X on-target coverage was obtained for the combined replicate libraries with 85% of on-target reads exceeding 100X depth.

Mutation Calling

Aligned and processed bam files were compared for calling somatic variants between treatments in a pairwise fashion using MuTect (1.1.4, GATK, Broad Institute). Similar comparisons for indels were done using GATK4 MuTect2 (2.1-beta, Broad Institute). Raw whole-exome sequencing (WES) FastQ data for patient-matched normal HCC1143-BL cell-lines, previously sequenced by Daemon, et al (2013)[3] and available on GEO under accession number GSE48216, was aligned using the pipeline described above and the resulting bam files were used as a "matched normal" in calling point mutations and indels in all three treatment pools vs. "normal" also using MuTect as above. All resulting variants were filtered to those labeled "KEEP" by MuTect and having at least 30X coverage and 10% variant allele frequency (vaf) in the "tumor" (treatment) and at least 15X coverage (with no presence of the alternate base) in the "normal" (HCC1143-BL, or second treatment for pairwise analysis). Variants listed in the dbSNP database (build 137, https://www.ncbi.nlm.nih.gov/projects/SNP/) were omitted and the resulting variant list was further hand curated using the Integrative Genomics Viewer (IGV, 2.3.82, http://software.broadinstitute.org/software/igv/) and SAMtools mpileup software[2] (1.2, https://github.com/samtools/samtools) to identify error-rich reads and other sources of errant calls. Variant allele frequencies presented for calls against HCC1143-BL were not corrected for differences in copy number or ploidy. Degenerate tags with sample-specific, fixed-indices introduced at the point of adaptor ligation were used to distinguish reads representing PCR

duplicates from reads mapping to the same start and stop position yet derived from unique input molecules, either from the same or independent library preparation(s), in order to preserve the high complexity of the combined treatment libraries observed when sequenced to high depth (in-house software).

References

1. Chapman, M. P. *et al.* A model of phenotypic state dynamics initiates a promising approach to control heterogeneous malignant cell populations. in *2016 IEEE 55th Conference on Decision and Control (CDC)* 2481–2487 (2016). doi:10.1109/CDC.2016.7798634
2. Li, H. *et al.* The Sequence Alignment/Map format and SAMtools. *Bioinforma. Oxf. Engl.* **25,** 2078–2079 (2009).
3. Daemen, A. *et al.* Modeling precision treatment of breast cancer. *Genome Biol.* **14,** R110 (2013).
4. Gusnanto, A., Wood, H. M., Pawitan, Y., Rabbitts, P. & Berri, S. Correcting for cancer genome size and tumour cell content enables better estimation of copy number alterations from next-generation sequence data. *Bioinformatics* **28,** 40–47 (2012).
