# Supplementary material for: Differentiation-state plasticity is a targetable resistance mechanism in basal-like breast cancer
Source: Nat Commun. 2018 Sep 19;9:3815. doi: 10.1038/s41467-018-05729-w (PMC6145927; doi:10.1038/s41467-018-05729-w)
Supplement: Supplementary file 3 — Description of Additional Supplementary Files [file 41467_2018_5729_MOESM3_ESM.pdf]

Description of Additional Supplementary Files

File Name: Supplementary Data 1
Description: Images and accompanying image cytometry analysis for all tumors analyzed in Figure 1

File Name: Supplementary Data 2
Description: Results from the targeted therapy screen in HCC1143 and SUM149PT cell lines

File Name: Supplementary Data 3
Description: Tables describing the genesets used in the study and all raw GSEA results

File Name: Supplementary Data 4
Description: A zip file containing all raw .vcf files from Mutect analyses and .seg files from LP-WGS copy number alteration analyses

File Name: Supplementary Data 5
Description: VIPER results and pathway ontology analyses

File Name: Supplementary Data 6
Description: SciATAC-seq DNA-binding domain motif enrichment analysis
