# Supplementary material for: Differentiation-state plasticity is a targetable resistance mechanism in basal-like breast cancer
Source: Nat Commun. 2018 Sep 19;9:3815. doi: 10.1038/s41467-018-05729-w (PMC6145927; doi:10.1038/s41467-018-05729-w)
Supplement: Supplementary file 4 — Supplementary Data 1 [file 41467_2018_5729_MOESM4_ESM.pdf]

Supplementary Data 1

Measuring differentiation-state heterogeneity in primary patient samples and patient-derived xenografts.

All patient tumors (p.2-7) and patient-derived xenograft tumors (p.8-11) analyzed in this study are presented with the following: 4-color IF images showing DAPI (yellow), K19 (blue), K14 (green) and VIM (red) with normal structures excluded, scale bars = 100μm (left panel). An x/y dotplot showing single cell location and cellular phenotype, with an accompanying legend showing phenotype counts (middle panel). Dot plots comparing single tumor cell intensity of K19 vs. VIM, and K14 vs. VIM, with positivity gates shown for each marker (right panel).

Triple Negative Tumors

IF: DAPI K19 K14 VIM

Cell Phenotype (K19 / K14 / VIM): + / - / - ; - / + / - ; - / - / + ; + / - / + ; + / + /- ; - / + / + ; + / + / + ; - / - / -

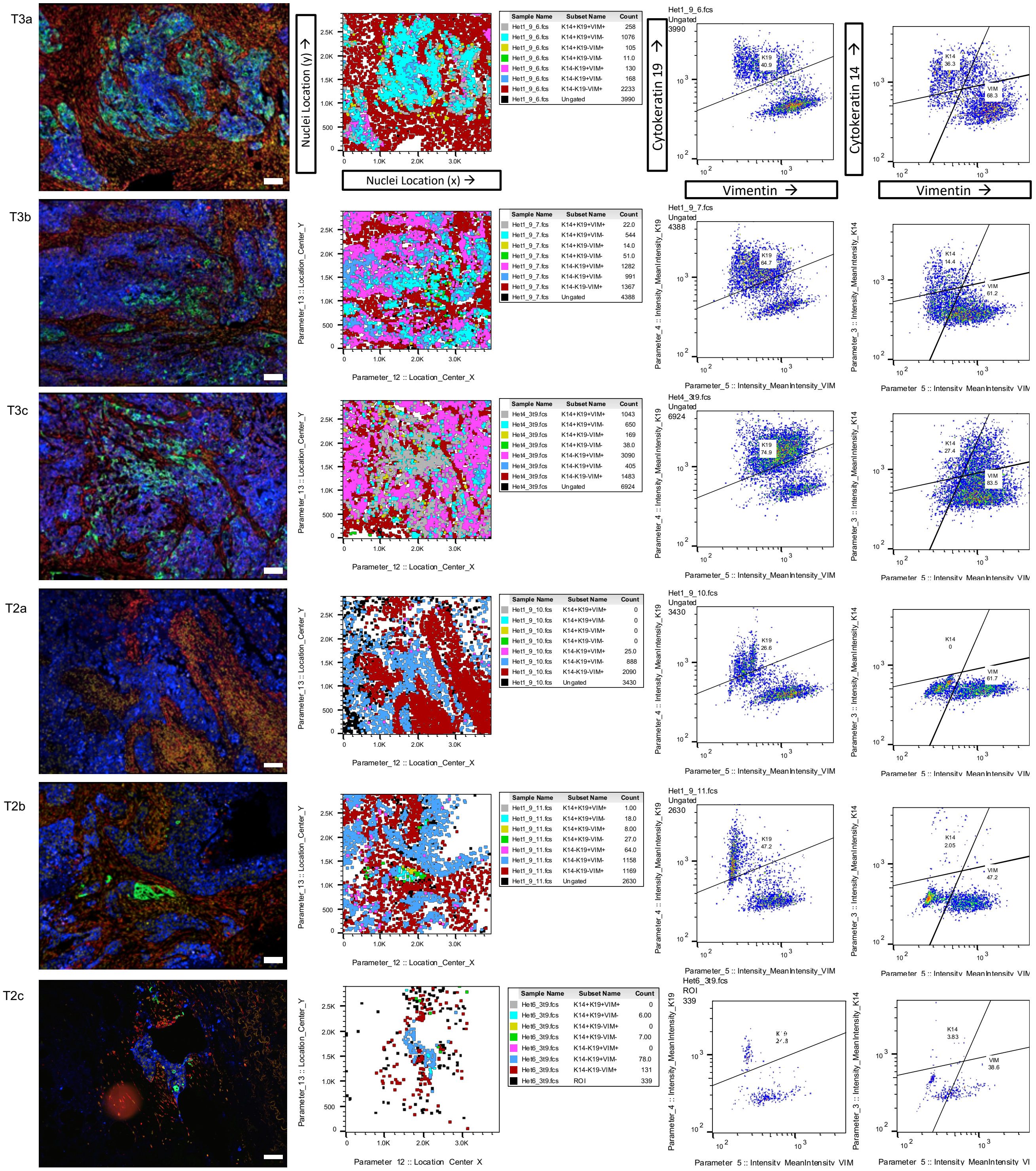

	Sample Name	Subset Name	Count
	Het1_9_6.fcs	K14+K19+VIM+	258
	Het1_9_6.fcs	K14+K19+VIM-	1076
	Het1_9_6.fcs	K14+K19-VIM+	105
	Het1_9_6.fcs	K14+K19-VIM-	11.0
	Het1_9_6.fcs	K14-K19+VIM+	130
	Het1_9_6.fcs	K14-K19+VIM-	168
	Het1_9_6.fcs	K14-K19-VIM+	2233
	Het1_9_6.fcs	Ungated	3990

	Sample Name	Subset Name	Count
	Het1_9_7.fcs	K14+K19+VIM+	22.0
	Het1_9_7.fcs	K14+K19+VIM-	544
	Het1_9_7.fcs	K14+K19-VIM+	14.0
	Het1_9_7.fcs	K14+K19-VIM-	51.0
	Het1_9_7.fcs	K14-K19+VIM+	1282
	Het1_9_7.fcs	K14-K19+VIM-	991
	Het1_9_7.fcs	K14-K19-VIM+	1367
	Het1_9_7.fcs	Ungated	4388

	Sample Name	Subset Name	Count
	Het4_3t9.fcs	K14+K19+VIM+	1043
	Het4_3t9.fcs	K14+K19+VIM-	650
	Het4_3t9.fcs	K14+K19-VIM+	169
	Het4_3t9.fcs	K14+K19-VIM-	38.0
	Het4_3t9.fcs	K14-K19+VIM+	3090
	Het4_3t9.fcs	K14-K19+VIM-	405
	Het4_3t9.fcs	K14-K19-VIM+	1483
	Het4_3t9.fcs	Ungated	6924

	Sample Name	Subset Name	Count
	Het1_9_10.fcs	K14+K19+VIM+	0
	Het1_9_10.fcs	K14+K19+VIM-	0
	Het1_9_10.fcs	K14+K19-VIM+	0
	Het1_9_10.fcs	K14+K19-VIM-	0
	Het1_9_10.fcs	K14-K19+VIM+	25.0
	Het1_9_10.fcs	K14-K19+VIM-	888
	Het1_9_10.fcs	K14-K19-VIM+	2090
	Het1_9_10.fcs	Ungated	3430

	Sample Name	Subset Name	Count
	Het1_9_11.fcs	K14+K19+VIM+	1.00
	Het1_9_11.fcs	K14+K19+VIM-	18.0
	Het1_9_11.fcs	K14+K19-VIM+	8.00
	Het1_9_11.fcs	K14+K19-VIM-	27.0
	Het1_9_11.fcs	K14-K19+VIM+	64.0
	Het1_9_11.fcs	K14-K19+VIM-	1158
	Het1_9_11.fcs	K14-K19-VIM+	1169
	Het1_9_11.fcs	Ungated	2630

	Sample Name	Subset Name	Count
	Het6_3t9.fcs	K14+K19+VIM+	0
	Het6_3t9.fcs	K14+K19+VIM-	6.00
	Het6_3t9.fcs	K14+K19-VIM+	0
	Het6_3t9.fcs	K14+K19-VIM-	7.00
	Het6_3t9.fcs	K14-K19+VIM+	0
	Het6_3t9.fcs	K14-K19+VIM-	78.0
	Het6_3t9.fcs	K14-K19-VIM+	131
	Het6_3t9.fcs	ROI	339

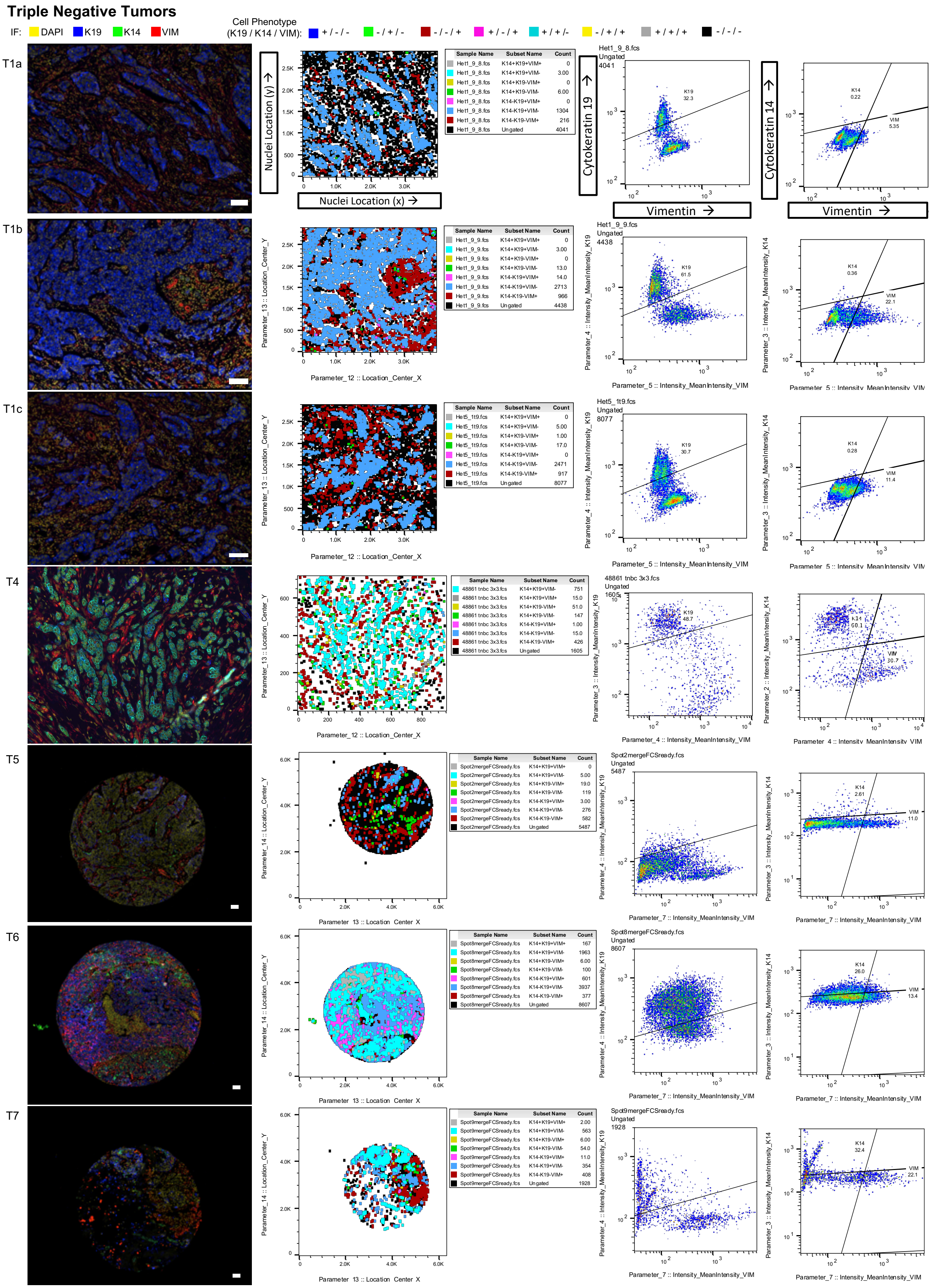

Triple Negative Tumors
IF: DAPI K19 K14 VIM
Cell Phenotype (K19 / K14 / VIM): + / - / - - / + / - - / - / + + / - / + + / + / - - / + / + + / + / + - / - / -
T1a
T1b
T1c
T4
T5
T6
T7
Nuclei Location (y)
Nuclei Location (x)
Cytokeratin 19
Cytokeratin 14
Vimentin
Parameter_12 :: Location_Center_X
Parameter_13 :: Location_Center_Y
Parameter_5 :: Intensity_MeanIntensity_VIM
Parameter_4 :: Intensity_MeanIntensity_K19
Parameter_3 :: Intensity_MeanIntensity_K14
Parameter_7 :: Intensity_MeanIntensity_VIM
Het1_9_8.fcs
Het1_9_9.fcs
Het5_1t9.fcs
48861 tnbc 3x3.fcs
Spot2mergeFCSready.fcs
Spot8mergeFCSready.fcs
Spot9mergeFCSready.fcs
Ungated
Sample Name
Subset Name
Count

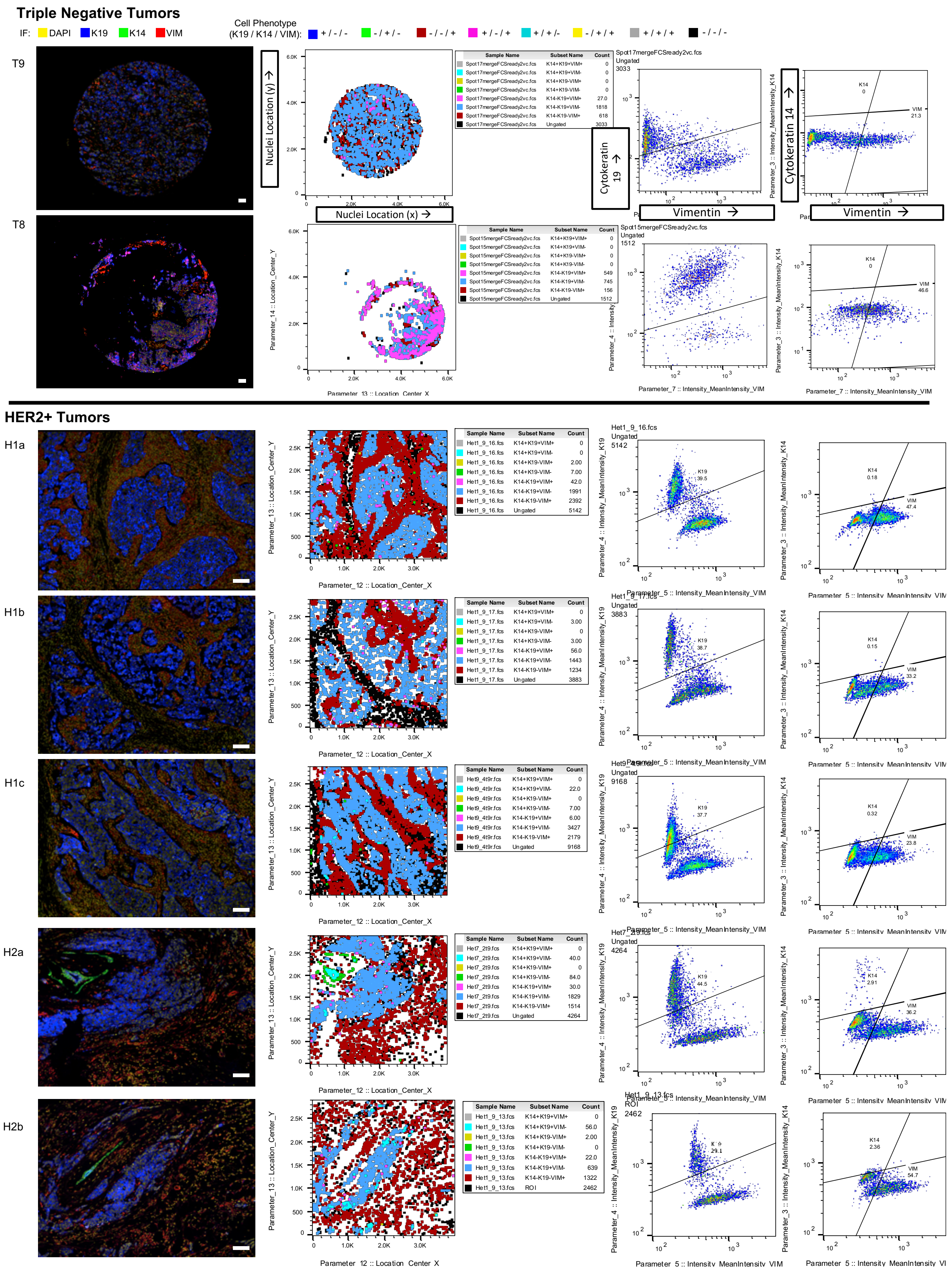

Triple Negative Tumors
IF: DAPI K19 K14 VIM
Cell Phenotype (K19 / K14 / VIM): + / - / - ; - / + / - ; - / - / + ; + / - / + ; + / + / - ; - / + / + ; + / + / + ; - / - / -
T9
T8
Nuclei Location (y) →
Nuclei Location (x) →
Cytokeratin 19 →
Cytokeratin 14 →
Vimentin →
Spot17mergeFCSready2vc.fcs
Ungated
3033
Spot15mergeFCSready2vc.fcs
Ungated
1512
Parameter_14 :: Location_Center_Y
Parameter_13 :: Location_Center_X
Parameter_4 :: Intensity_MeanIntensity_K19
Parameter_3 :: Intensity_MeanIntensity_K14
Parameter_7 :: Intensity_MeanIntensity_VIM
HER2+ Tumors
H1a
H1b
H1c
H2a
H2b
Parameter_13 :: Location_Center_Y
Parameter_12 :: Location_Center_X
Parameter_5 :: Intensity_MeanIntensity_VIM
Het1_9_16.fcs
Ungated
5142
Het1_9_17.fcs
Ungated
3883
Het9_4t9r.fcs
Ungated
9168
Het7_2t9.fcs
Ungated
4264
Het1_9_13.fcs
ROI
2462
Sample Name
Subset Name
Count

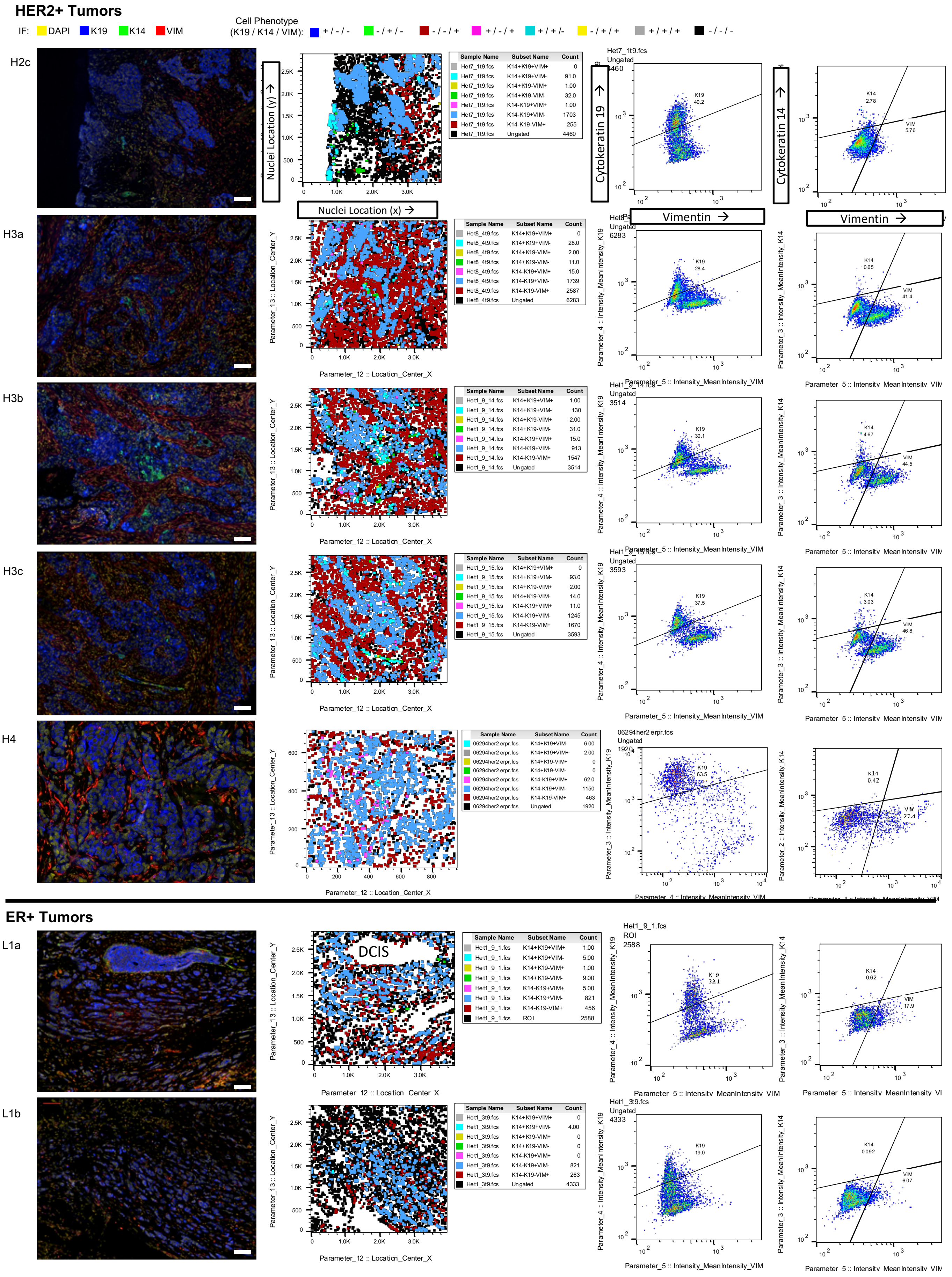
HER2+ Tumors
IF: DAPI K19 K14 VIM
Cell Phenotype (K19 / K14 / VIM): + / - / - ; - / + / - ; - / - / + ; + / - / + ; + / + /- ; - / + / + ; + / + / + ; - / - / -
H2c
H3a
H3b
H3c
H4
Nuclei Location (y)
Nuclei Location (x)
Cytokeratin 19
Cytokeratin 14
Vimentin
Parameter_12 :: Location_Center_X
Parameter_13 :: Location_Center_Y
Parameter_5 :: Intensity_MeanIntensity_VIM
Parameter_4 :: Intensity_MeanIntensity_K19
Parameter_3 :: Intensity_MeanIntensity_K14
ER+ Tumors
L1a
L1b
DCIS

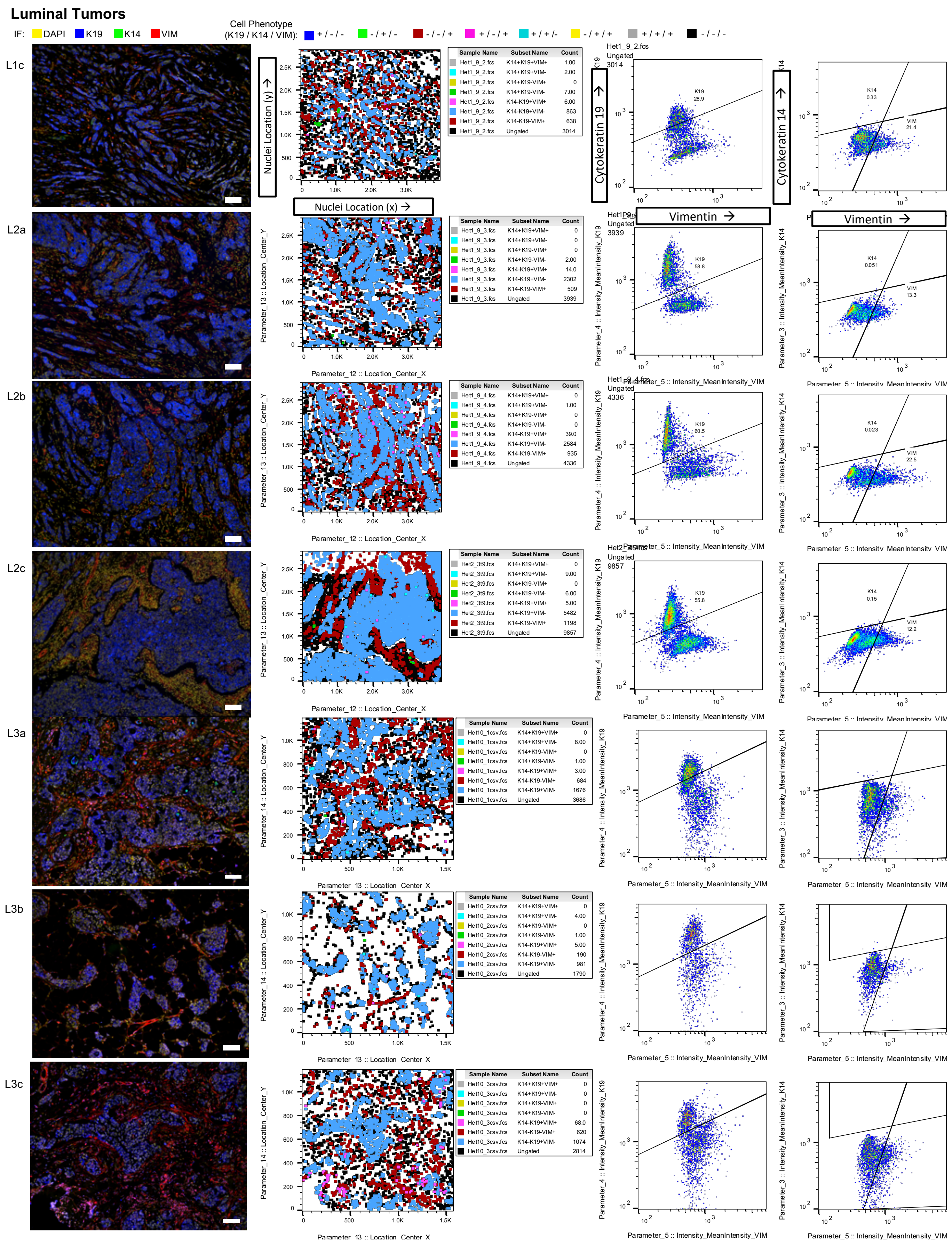

Luminal Tumors
IF: DAPI K19 K14 VIM
Cell Phenotype (K19 / K14 / VIM): + / - / - ; - / + / - ; - / - / + ; + / - / + ; + / + / - ; - / + / + ; + / + / + ; - / - / -
L1c
L2a
L2b
L2c
L3a
L3b
L3c
Nuclei Location (y)
Nuclei Location (x)
Cytokeratin 19
Cytokeratin 14
Vimentin
Sample Name
Subset Name
Count
Parameter_12 :: Location_Center_X
Parameter_13 :: Location_Center_Y
Parameter_5 :: Intensity_MeanIntensity_VIM
Parameter_4 :: Intensity_MeanIntensity_K19
Parameter_3 :: Intensity_MeanIntensity_K14

Luminal Tumors

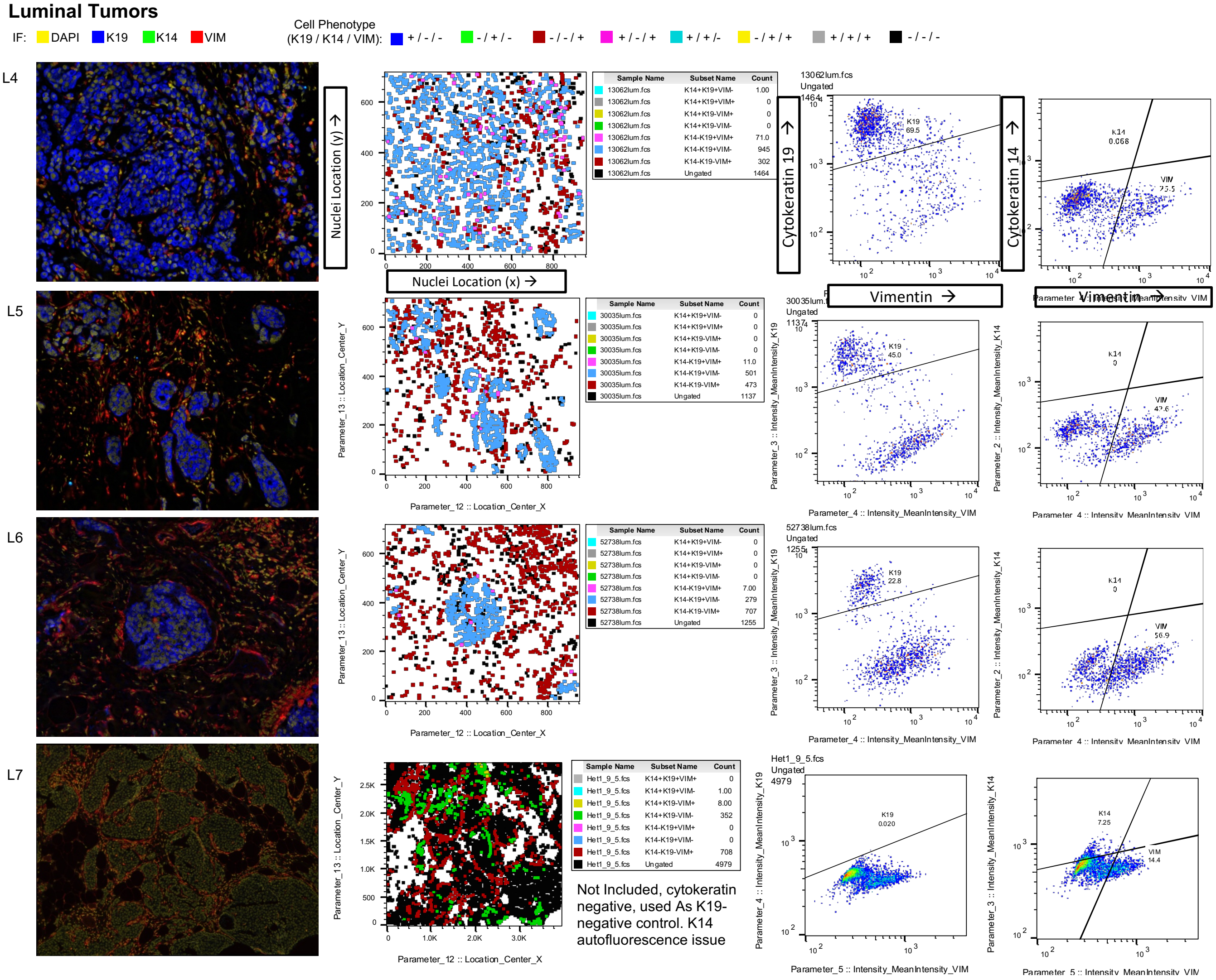

Sample Name	Subset Name	Count
13062lum.fcs	K14+K19+VIM-	1.00
13062lum.fcs	K14+K19+VIM+	0
13062lum.fcs	K14+K19-VIM+	0
13062lum.fcs	K14+K19-VIM-	0
13062lum.fcs	K14-K19+VIM+	71.0
13062lum.fcs	K14-K19+VIM-	945
13062lum.fcs	K14-K19-VIM+	302
13062lum.fcs	Ungated	1464

Sample Name	Subset Name	Count
30035lum.fcs	K14+K19+VIM-	0
30035lum.fcs	K14+K19+VIM+	0
30035lum.fcs	K14+K19-VIM+	0
30035lum.fcs	K14+K19-VIM-	0
30035lum.fcs	K14-K19+VIM+	11.0
30035lum.fcs	K14-K19+VIM-	501
30035lum.fcs	K14-K19-VIM+	473
30035lum.fcs	Ungated	1137

Sample Name	Subset Name	Count
52738lum.fcs	K14+K19+VIM-	0
52738lum.fcs	K14+K19+VIM+	0
52738lum.fcs	K14+K19-VIM+	0
52738lum.fcs	K14+K19-VIM-	0
52738lum.fcs	K14-K19+VIM+	7.00
52738lum.fcs	K14-K19+VIM-	279
52738lum.fcs	K14-K19-VIM+	707
52738lum.fcs	Ungated	1255

Sample Name	Subset Name	Count
Het1_9_5.fcs	K14+K19+VIM+	0
Het1_9_5.fcs	K14+K19+VIM-	1.00
Het1_9_5.fcs	K14+K19-VIM+	8.00
Het1_9_5.fcs	K14+K19-VIM-	352
Het1_9_5.fcs	K14-K19+VIM+	0
Het1_9_5.fcs	K14-K19+VIM-	0
Het1_9_5.fcs	K14-K19-VIM+	708
Het1_9_5.fcs	Ungated	4979

Not Included, cytokeratin negative, used As K19-negative control. K14 autofluorescence issue

Patient-Derived Xenograft Tumors

IF: DAPI, K19, K14, VIM, Ku80

Ku80+ Tumor Cell Phenotype (K19 / K14 / VIM): + / - / -, - / + / -, - / - / +, + / - / +, + / + /-, - / + / +, + / + / +; Ku80-negative cell

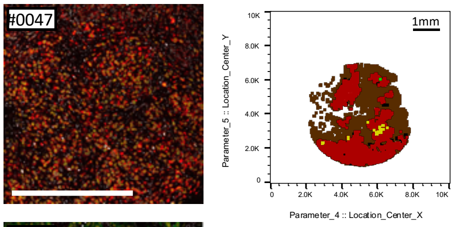

Sample Name	Subset Name	Count
MultispotimagesMERGE_2.fcs	K14+K19+VIM+	0
MultispotimagesMERGE_2.fcs	K14+K19+VIM-	0
MultispotimagesMERGE_2.fcs	K14+K19-VIM+	0
MultispotimagesMERGE_2.fcs	K14+K19-VIM-	0
MultispotimagesMERGE_2.fcs	K14-K19+VIM+	0
MultispotimagesMERGE_2.fcs	K14-K19+VIM-	0
MultispotimagesMERGE_2.fcs	K14-K19-VIM+	0
MultispotimagesMERGE_2.fcs	TumorCell	4202
MultispotimagesMERGE_2.fcs	Ungated	24707

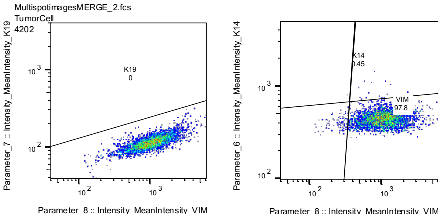

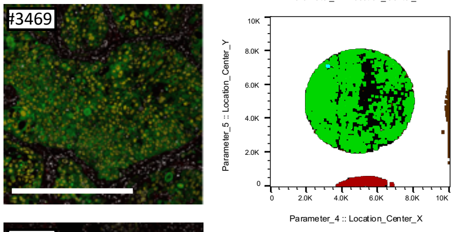

Sample Name	Subset Name	Count
MultispotimagesMERGE_3.fcs	K14+K19+VIM+	0
MultispotimagesMERGE_3.fcs	K14+K19+VIM-	2.00
MultispotimagesMERGE_3.fcs	K14+K19-VIM+	0
MultispotimagesMERGE_3.fcs	K14+K19-VIM-	4473
MultispotimagesMERGE_3.fcs	K14-K19+VIM+	0
MultispotimagesMERGE_3.fcs	K14-K19+VIM-	1.00
MultispotimagesMERGE_3.fcs	K14-K19-VIM+	795
MultispotimagesMERGE_3.fcs	TumorCell	27611
MultispotimagesMERGE_3.fcs	Ungated	82028

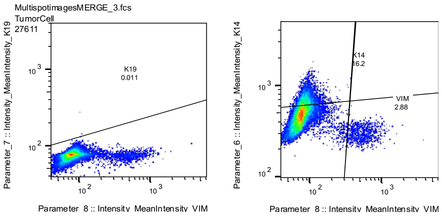

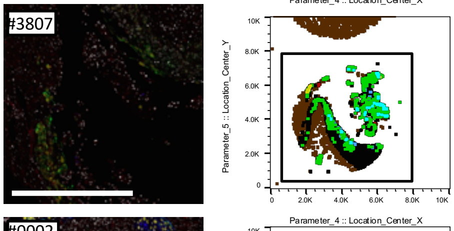

Sample Name	Subset Name	Count
MultispotimagesMERGE_4.fcs	K14+K19+VIM+	0
MultispotimagesMERGE_4.fcs	K14+K19+VIM-	55.0
MultispotimagesMERGE_4.fcs	K14+K19-VIM+	8.00
MultispotimagesMERGE_4.fcs	K14+K19-VIM-	975
MultispotimagesMERGE_4.fcs	K14-K19+VIM+	0
MultispotimagesMERGE_4.fcs	K14-K19+VIM-	65.0
MultispotimagesMERGE_4.fcs	K14-K19-VIM+	17.0
MultispotimagesMERGE_4.fcs	TumorCell	8102
MultispotimagesMERGE_4.fcs	Ungated	98767

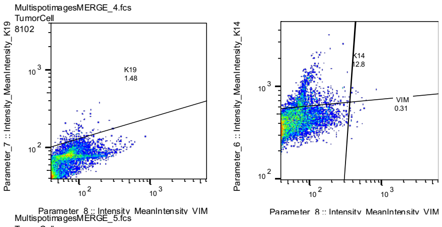

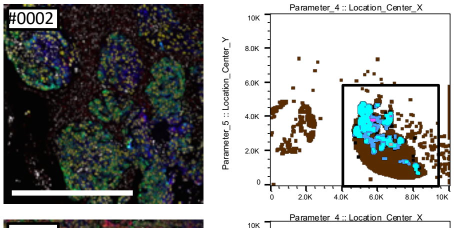

Sample Name	Subset Name	Count
MultispotimagesMERGE_5.fcs	K14+K19+VIM+	0
MultispotimagesMERGE_5.fcs	K14+K19+VIM-	0
MultispotimagesMERGE_5.fcs	K14+K19-VIM+	0
MultispotimagesMERGE_5.fcs	K14+K19-VIM-	0
MultispotimagesMERGE_5.fcs	K14-K19+VIM+	0
MultispotimagesMERGE_5.fcs	K14-K19+VIM-	0
MultispotimagesMERGE_5.fcs	K14-K19-VIM+	0
MultispotimagesMERGE_5.fcs	TumorCell	2431
MultispotimagesMERGE_5.fcs	Ungated	19151

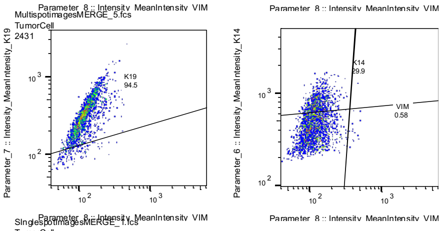

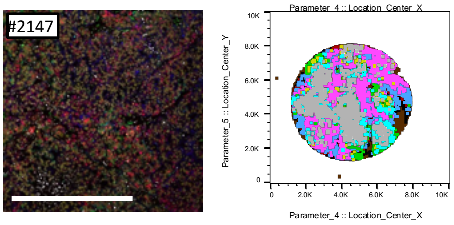

Sample Name	Subset Name	Count
SInglespotImagesMERGE_1.fcs	K14+K19+VIM+	2355
SInglespotImagesMERGE_1.fcs	K14+K19+VIM-	2844
SInglespotImagesMERGE_1.fcs	K14+K19-VIM+	610
SInglespotImagesMERGE_1.fcs	K14+K19-VIM-	2411
SInglespotImagesMERGE_1.fcs	K14-K19+VIM+	8242
SInglespotImagesMERGE_1.fcs	K14-K19+VIM-	9386
SInglespotImagesMERGE_1.fcs	K14-K19-VIM+	2460
SInglespotImagesMERGE_1.fcs	TumorCell	36091
SInglespotImagesMERGE_1.fcs	Ungated	70208

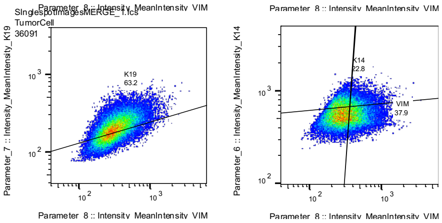

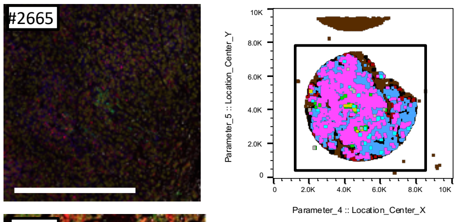

Sample Name	Subset Name	Count
SInglespotImagesMERGE_2.fcs	K14+K19+VIM+	9.00
SInglespotImagesMERGE_2.fcs	K14+K19+VIM-	35.0
SInglespotImagesMERGE_2.fcs	K14+K19-VIM+	13.0
SInglespotImagesMERGE_2.fcs	K14+K19-VIM-	34.0
SInglespotImagesMERGE_2.fcs	K14-K19+VIM+	2553
SInglespotImagesMERGE_2.fcs	K14-K19+VIM-	8033
SInglespotImagesMERGE_2.fcs	K14-K19-VIM+	1340
SInglespotImagesMERGE_2.fcs	TumorCell	28043
SInglespotImagesMERGE_2.fcs	Ungated	52107

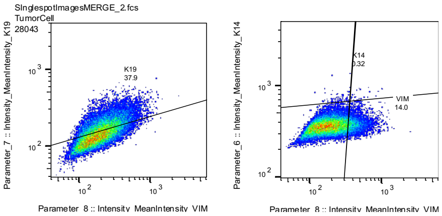

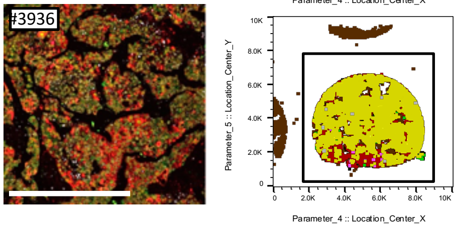

Sample Name	Subset Name	Count
SInglespotImagesMERGE_4.fcs	K14+K19+VIM+	7.00
SInglespotImagesMERGE_4.fcs	K14+K19+VIM-	0
SInglespotImagesMERGE_4.fcs	K14+K19-VIM+	9911
SInglespotImagesMERGE_4.fcs	K14+K19-VIM-	110
SInglespotImagesMERGE_4.fcs	K14-K19+VIM+	31.0
SInglespotImagesMERGE_4.fcs	K14-K19+VIM-	1.00
SInglespotImagesMERGE_4.fcs	K14-K19-VIM+	16023
SInglespotImagesMERGE_4.fcs	TumorCell	26492
SInglespotImagesMERGE_4.fcs	Ungated	47868

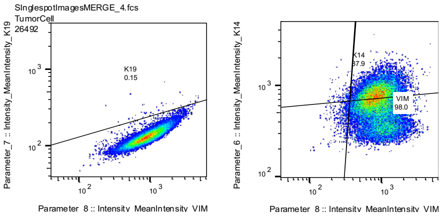

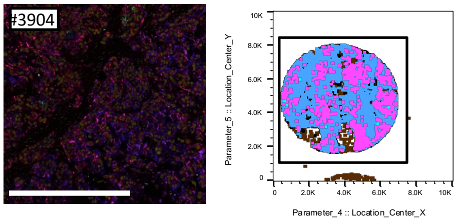

Sample Name	Subset Name	Count
SInglespotImagesMERGE_20.fcs	K14+K19+VIM+	0
SInglespotImagesMERGE_20.fcs	K14+K19+VIM-	0
SInglespotImagesMERGE_20.fcs	K14+K19-VIM+	0
SInglespotImagesMERGE_20.fcs	K14+K19-VIM-	0
SInglespotImagesMERGE_20.fcs	K14-K19+VIM+	0
SInglespotImagesMERGE_20.fcs	K14-K19+VIM-	0
SInglespotImagesMERGE_20.fcs	K14-K19-VIM+	0
SInglespotImagesMERGE_20.fcs	TumorCell	10253
SInglespotImagesMERGE_20.fcs	Ungated	24594

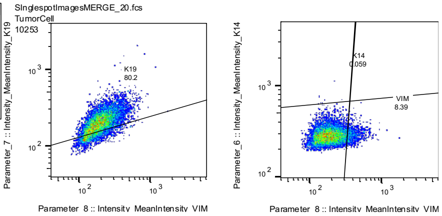

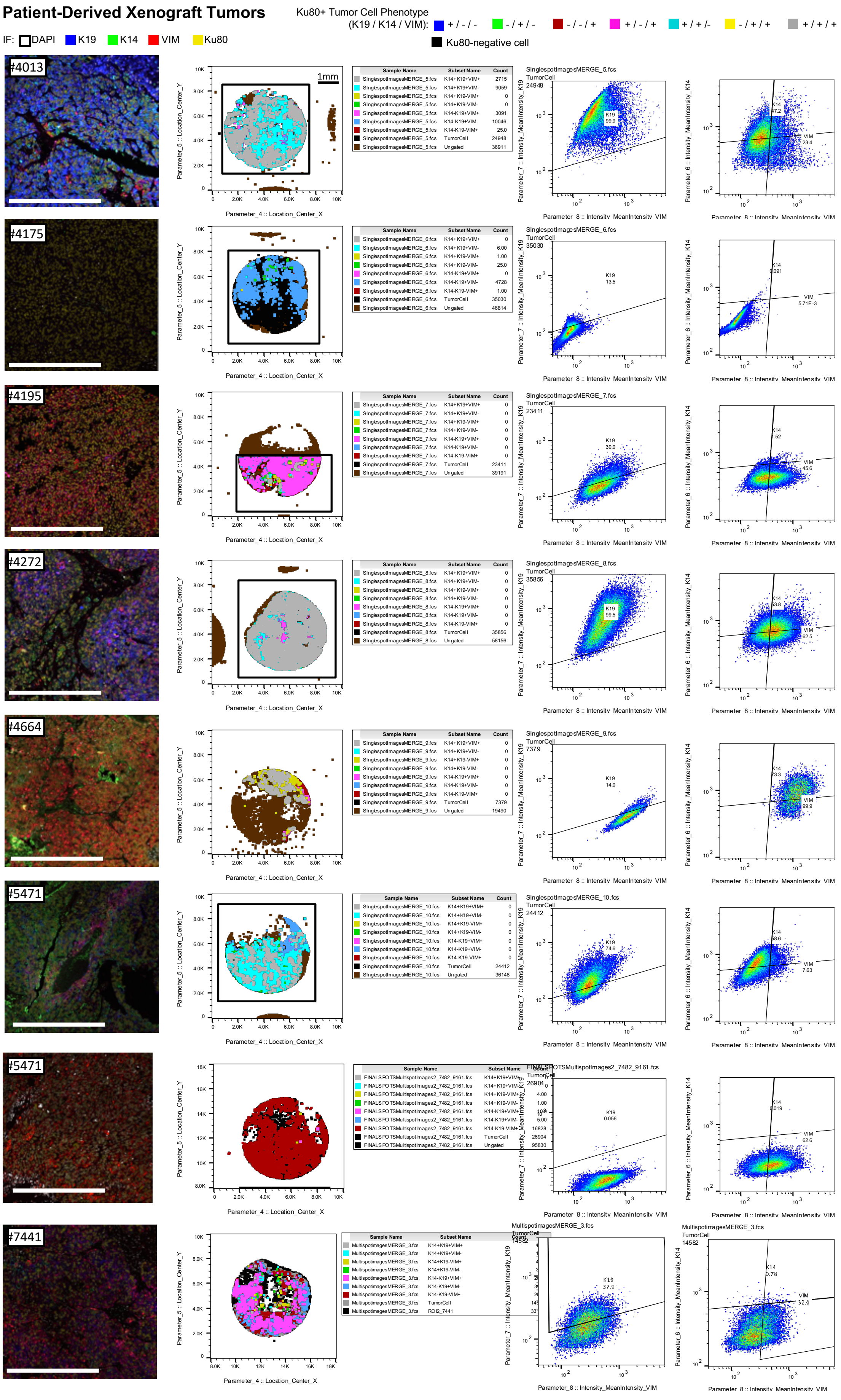
Patient-Derived Xenograft Tumors
IF: DAPI K19 K14 VIM Ku80
Ku80+ Tumor Cell Phenotype
(K19 / K14 / VIM): + / - / - ; - / + / - ; - / - / + ; + / - / + ; + / + /- ; - / + / + ; + / + / +
Ku80-negative cell
#4013
#4175
#4195
#4272
#4664
#5471
#5471
#7441
1mm
Parameter_4 :: Location_Center_X
Parameter_5 :: Location_Center_Y
Parameter_7 :: Intensity_MeanIntensity_K19
Parameter_6 :: Intensity_MeanIntensity_K14
Parameter_8 :: Intensity_MeanIntensity_VIM

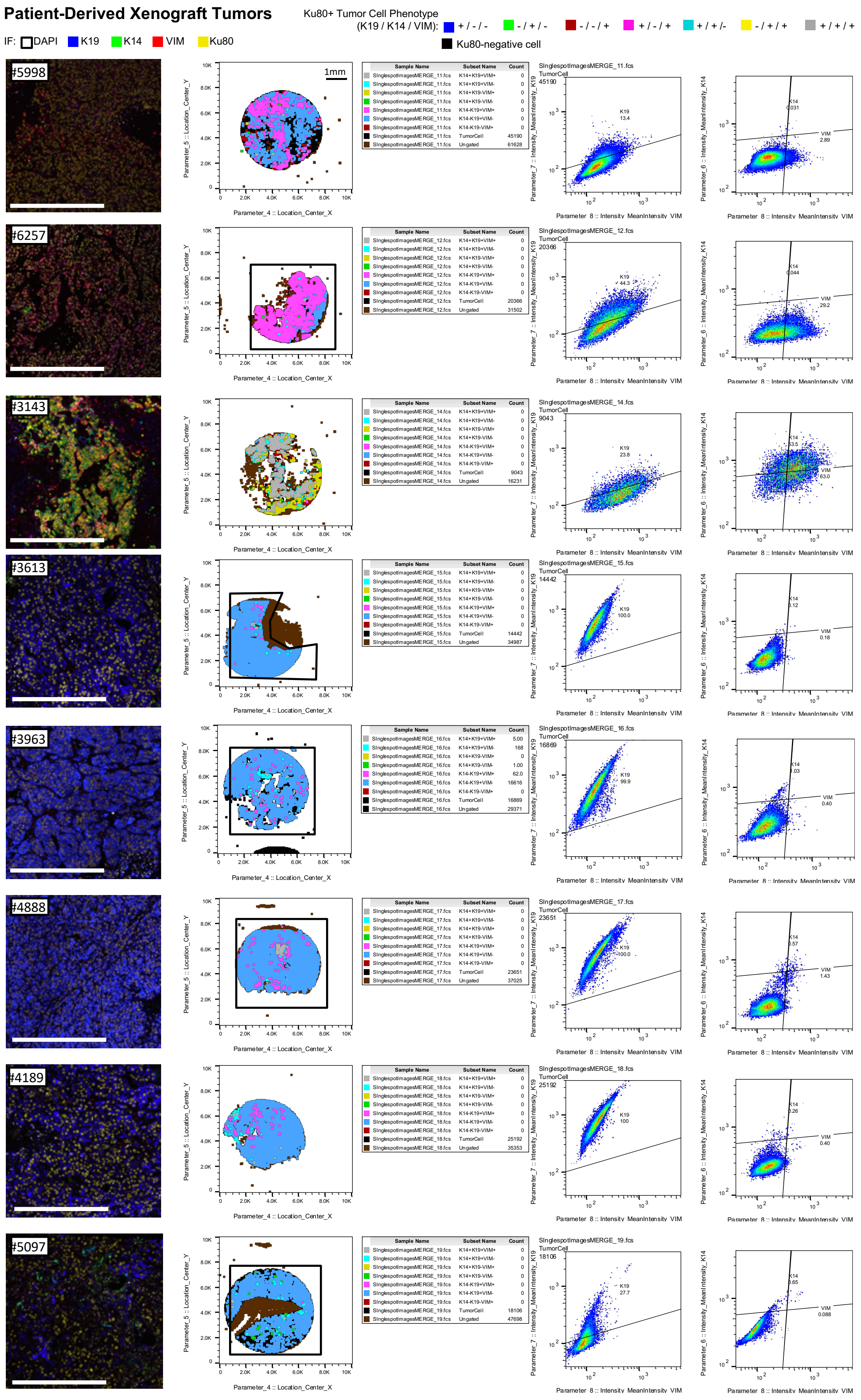

Patient-Derived Xenograft Tumors
IF: DAPI K19 K14 VIM Ku80
Ku80+ Tumor Cell Phenotype
(K19 / K14 / VIM):
+ / - / -
- / + / -
- / - / +
+ / - / +
+ / + /-
- / + / +
+ / + / +
Ku80-negative cell
#5998
#6257
#3143
#3613
#3963
#4888
#4189
#5097
1mm
Parameter_4 :: Location_Center_X
Parameter_5 :: Location_Center_Y
Sample Name | Subset Name | Count
SInglespotImagesMERGE_11.fcs K14+K19+VIM+ 0; K14+K19+VIM- 0; K14+K19-VIM+ 0; K14+K19-VIM- 0; K14-K19+VIM+ 0; K14-K19+VIM- 0; K14-K19-VIM+ 0; TumorCell 45190; Ungated 61628
SInglespotImagesMERGE_12.fcs K14+K19+VIM+ 0; K14+K19+VIM- 0; K14+K19-VIM+ 0; K14+K19-VIM- 0; K14-K19+VIM+ 0; K14-K19+VIM- 0; K14-K19-VIM+ 0; TumorCell 20366; Ungated 31502
SInglespotImagesMERGE_14.fcs K14+K19+VIM+ 0; K14+K19+VIM- 0; K14+K19-VIM+ 0; K14+K19-VIM- 0; K14-K19+VIM+ 0; K14-K19+VIM- 0; K14-K19-VIM+ 0; TumorCell 9043; Ungated 16231
SInglespotImagesMERGE_15.fcs K14+K19+VIM+ 0; K14+K19+VIM- 0; K14+K19-VIM+ 0; K14+K19-VIM- 0; K14-K19+VIM+ 0; K14-K19+VIM- 0; K14-K19-VIM+ 0; TumorCell 14442; Ungated 34987
SInglespotImagesMERGE_16.fcs K14+K19+VIM+ 5.00; K14+K19+VIM- 168; K14+K19-VIM+ 0; K14+K19-VIM- 1.00; K14-K19+VIM+ 62.0; K14-K19+VIM- 16616; K14-K19-VIM+ 0; TumorCell 16869; Ungated 29371
SInglespotImagesMERGE_17.fcs K14+K19+VIM+ 0; K14+K19+VIM- 0; K14+K19-VIM+ 0; K14+K19-VIM- 0; K14-K19+VIM+ 0; K14-K19+VIM- 0; K14-K19-VIM+ 0; TumorCell 23651; Ungated 37025
SInglespotImagesMERGE_18.fcs K14+K19+VIM+ 0; K14+K19+VIM- 0; K14+K19-VIM+ 0; K14+K19-VIM- 0; K14-K19+VIM+ 0; K14-K19+VIM- 0; K14-K19-VIM+ 0; TumorCell 25192; Ungated 35353
SInglespotImagesMERGE_19.fcs K14+K19+VIM+ 0; K14+K19+VIM- 0; K14+K19-VIM+ 0; K14+K19-VIM- 0; K14-K19+VIM+ 0; K14-K19+VIM- 0; K14-K19-VIM+ 0; TumorCell 18106; Ungated 47698
SInglespotImagesMERGE_11.fcs TumorCell 45190 K19 13.4 K14 0.031 VIM 2.89
SInglespotImagesMERGE_12.fcs TumorCell 20366 K19 44.3 K14 0.044 VIM 29.2
SInglespotImagesMERGE_14.fcs TumorCell 9043 K19 23.8 K14 33.5 VIM 63.0
SInglespotImagesMERGE_15.fcs TumorCell 14442 K19 100.0 K14 0.12 VIM 0.18
SInglespotImagesMERGE_16.fcs TumorCell 16869 K19 99.9 K14 1.03 VIM 0.40
SInglespotImagesMERGE_17.fcs TumorCell 23651 K19 100.0 K14 0.57 VIM 1.43
SInglespotImagesMERGE_18.fcs TumorCell 25192 K19 100 K14 0.26 VIM 0.40
SInglespotImagesMERGE_19.fcs TumorCell 18106 K19 27.7 K14 0.65 VIM 0.088
Parameter_7 :: Intensity_MeanIntensity_K19
Parameter_6 :: Intensity_MeanIntensity_K14
Parameter_8 :: Intensity_MeanIntensity_VIM

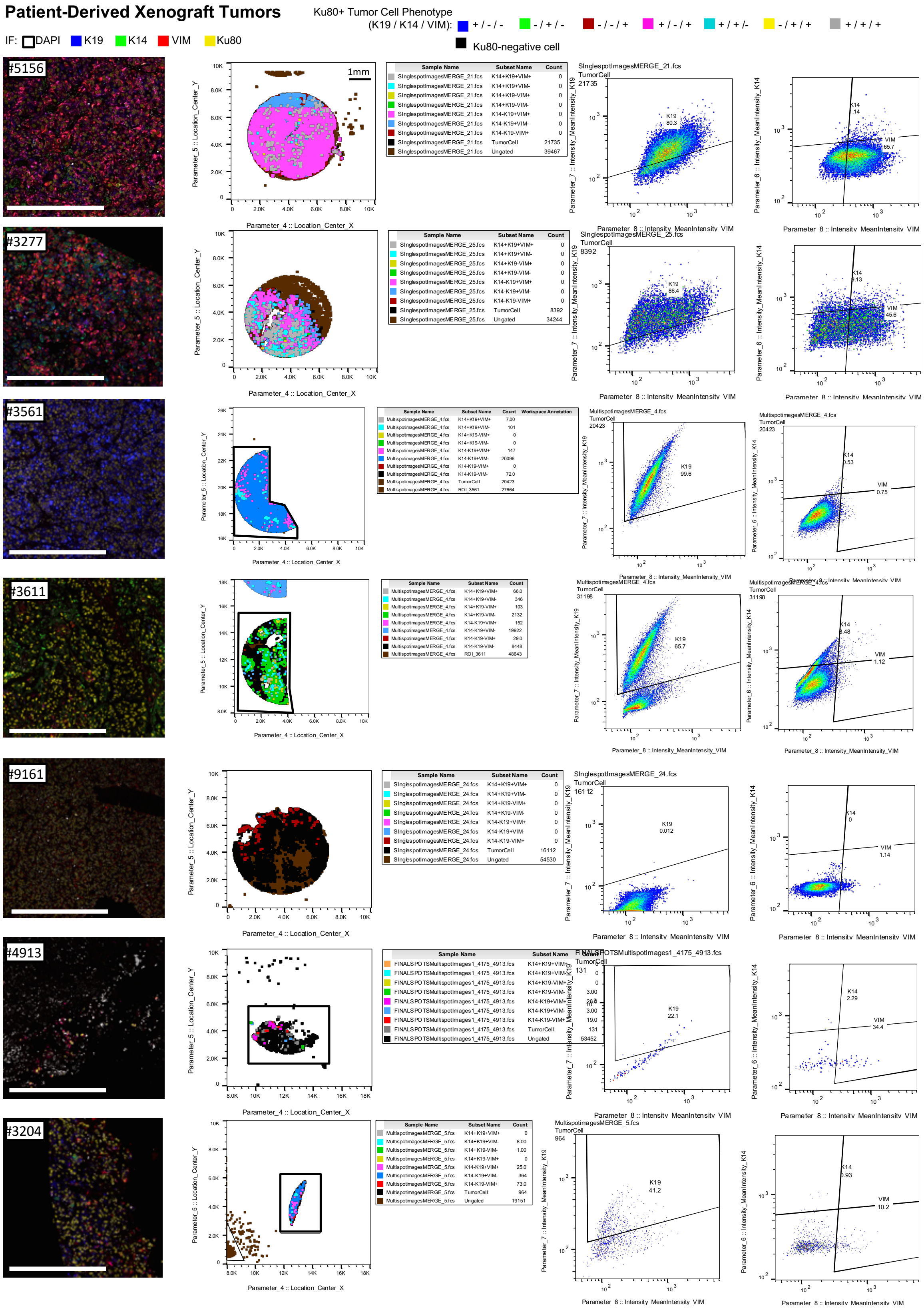

Patient-Derived Xenograft Tumors
IF: DAPI K19 K14 VIM Ku80
Ku80+ Tumor Cell Phenotype
(K19 / K14 / VIM):
+ / - / -
- / + / -
- / - / +
+ / - / +
+ / + / -
- / + / +
+ / + / +
Ku80-negative cell
#5156
#3277
#3561
#3611
#9161
#4913
#3204
1mm
Parameter_4 :: Location_Center_X
Parameter_5 :: Location_Center_Y
Parameter_8 :: Intensity_MeanIntensity_VIM
Parameter_7 :: Intensity_MeanIntensity_K19
Parameter_6 :: Intensity_MeanIntensity_K14
Sample Name
Subset Name
Count
SinglespotImagesMERGE_21.fcs
TumorCell 21735
Ungated 39467
K19 80.3
K14 1.14
VIM 65.7
SinglespotImagesMERGE_25.fcs
TumorCell 8392
Ungated 34244
K19 86.4
K14 0.13
VIM 45.6
MultispotImagesMERGE_4.fcs
TumorCell 20423
ROI_3561 27664
K19 99.6
K14 0.53
VIM 0.75
TumorCell 31198
ROI_3611 48643
K19 65.7
K14 3.48
VIM 1.12
SinglespotImagesMERGE_24.fcs
TumorCell 16112
Ungated 54530
K19 0.012
K14 0
VIM 1.14
FINALSPOTSMultispotImages1_4175_4913.fcs
TumorCell 131
Ungated 53452
K19 22.1
K14 2.29
VIM 34.4
MultispotImagesMERGE_5.fcs
TumorCell 964
Ungated 19151
K19 41.2
K14 0.93
VIM 10.2
